# Neuroprotective effects of cell-free supernatant from *Pediococcus pentosaceus* TAP041 against glycation- and inflammation-associated stress responses

**DOI:** 10.1007/s00253-026-13879-x

**Published:** 2026-05-22

**Authors:** Huijin Jeong, Young Seo Jang, Chaeeun Lee, Hak-Jong Choi, Young-Seo Park

**Affiliations:** 1 Department of Food Science and Biotechnology, Gachon University, Seongnam, 13120 Republic of Korea; 2 Technology Innovation Research Division, World Institute of Kimchi, Gwangju, 61755 Republic of Korea; 3 Department of Biotechnology, Graduate School, Korea University, Seoul, 02841 Republic of Korea

**Keywords:** Advanced glycation end products, Probiotics, *Pediococcus pentosaceus*, Neuroprotection, Safety assessment, Whole-genome sequencing

## Abstract

**Abstract:**

Neurodegenerative diseases are increasingly associated with the convergence of oxidative, inflammatory, and glycation-associated stress pathways. The present study evaluated the stress-modulatory activity and probiotic-related characteristics of *Pediococcus pentosaceus* TAP041, a lactic acid bacterium isolated from citrus fruit. cell-free supernatant from *P. pentosaceus* TAP041 (TAP041 CFS) was assessed in in vitro models of neuronal and glial stress. In SH-SY5Y neuroblastoma cells, TAP041 CFS was associated with improved cell viability, reduced intracellular ROS accumulation, a lower Bax/Bcl-2 mRNA ratio, and increased BDNF and TH mRNA expression under methylglyoxal (MGO)- and lipopolysaccharide (LPS)-induced stress. The protective effects under *N*^ε^-(carboxymethyl)-lysine (CML) exposure were comparatively limited, with partial modulation of apoptosis-related markers in the absence of significant viability recovery. In primary rat glial cells, TAP041 CFS attenuated LPS-induced GFAP immunoreactivity and suppressed IL-6 and TNF-α expression at the transcriptional level. TAP041 CFS also exhibited ABTS radical scavenging activity, providing direct biochemical evidence for secreted antioxidant constituents. The strain demonstrated gastrointestinal stress tolerance, intestinal adhesion capacity, and a favourable safety profile, including non-hemolytic and non-cytotoxic phenotypes, absence of transferable antibiotic resistance determinants, and no known virulence factors. whole-genome sequencing identified predicted pathways for glyoxalase-mediated detoxification, antioxidant defence, and B-vitamin biosynthesis, consistent with but not directly establishing the observed phenotypes. These findings identify *P. pentosaceus* TAP041 as a candidate strain warranting further investigation and provide a basis for future mechanistic and in vivo studies on probiotic-derived metabolites in neurodegeneration-related stress.

**Key points:**

• *TAP041 CFS reduced ROS, normalized Bax/Bcl-2, and suppressed glial inflammation.*

• *TAP041 exhibits probiotic potential with GI tolerance, adhesion, and a safe profile.*

• *Genome predicts glyoxalase and antioxidant pathways; ABTS confirms CFS antioxidants.*

**Supplementary Information:**

The online version contains supplementary material available at 10.1007/s00253-026-13879-x.

## Introduction

Neurodegenerative diseases (NDs) such as Alzheimer’s disease and Parkinson’s disease are characterised by progressive neuronal loss, resulting in cognitive and motor dysfunction. Although multifactorial in origin, oxidative stress, chronic neuroinflammation, protein misfolding, and mitochondrial dysfunction are the central contributors to disease pathogenesis (Liu et al. 2015). Among these, oxidative stress caused by an imbalance between reactive oxygen species (ROS) production and antioxidant defence mechanisms is particularly detrimental to neurons, which are highly susceptible because of the brain’s high oxygen demand and limited antioxidant capacity (Halliwell 1992).

A major upstream trigger of oxidative and inflammatory injury in NDs is the accumulation of advanced glycation end products (AGEs), a group of heterogeneous compounds formed by the non-enzymatic glycation of proteins, lipids, and nucleic acids. These include fluorescent species like pentosidine and non-fluorescent molecules such as *N*^ε^-(carboxymethyl)-lysine (CML), which are prevalent in both food and human tissues (Gaens et al. 2013). AGE precursors, such as methylglyoxal (MGO) and glyoxal (GO), which are generated during Maillard reactions and lipid peroxidation, exacerbate the AGE burden. Following ingestion, only 10–30% of AGEs are absorbed or excreted, whereas the remainder accumulate in tissues or reach the colon (Hellwig et al. 2019; Li et al. 2022).

AGEs bind to the receptor for advanced glycation end products (RAGE) and activate pro-oxidant and pro-inflammatory signaling pathways that contribute to neuronal damage (Jeong et al. 2020; Kislinger et al. 1999). MGO accumulation induces neuronal apoptosis and impairs cognitive function in animal models (Sena et al. 2012; Suh et al. 2014). Moreover, chronic AGE–RAGE activation is implicated in the development of metabolic and cardiovascular comorbidities (Bettiga et al. 2019; Gaens et al. 2014). These findings underscore the need for functional dietary agents capable of detoxifying AGE precursors and mitigating AGE-induced cellular damage.

The gut–brain axis, a bidirectional communication system between the central nervous system and gastrointestinal tract, regulates brain health and neuroinflammation (Kumar et al. 2025; Loh et al. 2024). Dysbiosis and compromised intestinal barrier function can amplify systemic oxidative and inflammatory stress and potentially exacerbate neurodegeneration (Aburto and Cryan 2024). Accordingly, probiotic interventions that modulate gut microbiota are candidate strains warranting strategies to counter AGE-induced neuronal stress via antioxidant, anti-inflammatory, and immunomodulatory mechanisms.

*Pediococcus pentosaceus* TAP041, isolated from tangerine (*Citrus reticulata* Blanco), exhibits notable MGO and GO detoxification capacities as well as tolerance to acidic and bile conditions (Lee and Park 2024). It also demonstrates strong adhesion to intestinal epithelial cells and elicits immunostimulatory activity through upregulation of iNOS, TNF-α, and IL-6, as well as MAPK pathway activation. These features support further evaluation of *P. pentosaceus* TAP041 as a probiotic candidate. Therefore, the present study aimed to provide an integrated characterisation of *P. pentosaceus* TAP041 using in vitro functional assays and genome analysis, rather than direct mechanistic confirmation. This integrated approach provides an integrated characterisation of *P. pentosaceus* TAP041 and offers preliminary support for further evaluation of its functional role in stress responses relevant to neurodegeneration.

## Materials and methods

### Preparation of LAB-derived cell-free supernatants

Three LAB strains were used: *Levilactobacillus brevis* KGK002 (KCCP 11467) and *P. pentosaceus* KCA007 (KCCP 11408), both isolated from kimchi, and *P. pentosaceus* TAP041 (KCCP 11195), isolated from *Citrus reticulata Blanco*. All strains were obtained from the Korean Culture Collection of Probiotics (KCCP, Seongnam, Korea). Each strain was routinely cultured in MRS broth (BD Biosciences, Franklin Lakes, NJ, USA) under anaerobic conditions at 37 °C for 16 h. A 1% (v/v) inoculum was then transferred to 50 mL of fresh MRS broth and incubated under the same conditions until the cultures reached the mid-logarithmic growth phase, corresponding to approximately 1 × 10^8^ CFU/mL.

To minimize carryover of residual MRS components, bacterial cells were harvested by centrifugation at 16,000 × g for 1 min at 4 °C, washed three times with sterile Dulbecco’s phosphate-buffered saline (DPBS; WELGENE, Gyeongsan, Korea), and resuspended in serum-free DMEM/F12 medium (1:1; HyClone; Cytiva, Marlborough, MA, USA) to a final density of 1 × 10^9^ CFU/mL. The bacterial suspensions were incubated at 37 °C for 24 h in a humidified atmosphere containing 5% CO_2_ to allow secretion of soluble metabolites into the mammalian cell culture medium. After incubation, the suspensions were centrifuged at 20,000 × g for 5 min at 4 °C, and the resulting cell-free supernatants (CFSs) were collected. The pH of each CFS was adjusted to 7.2–7.4 using sterile 5 N NaOH, and the samples were sterilized by filtration through a 0.22-μm PVDF syringe filter (Millipore, Burlington, MA, USA). Control medium was prepared in parallel using serum-free DMEM/F12 medium that was incubated under identical conditions without bacterial inoculation, followed by the same pH adjustment and filtration procedures. Freshly prepared CFSs were aliquoted and stored at − 80 °C for no longer than 8 weeks until use; repeated freeze–thaw cycles were avoided, and each aliquot was thawed only once. The working concentration of TAP041 CFS used in subsequent experiments was selected based on preliminary cell viability screening, which confirmed the absence of detectable cytotoxicity under the experimental conditions.

### Glial cell experiments

#### Primary glial cell culture and inflammation induction

All animal procedures were conducted in accordance with the guidelines of the U.S. National Institutes of Health for the care and use of laboratory animals. The experimental protocol was approved by the Institutional Animal Care and Use Committee of the World Institute of Kimchi (WIKIM IACUC 202426; Gwangju, Korea). Primary astrocytes were isolated from the cerebral cortices of postnatal day 2 Sprague–Dawley rat pups (OrientBio, Seongnam, Korea) following the method described by Woo et al. (2012) with minor modifications. Briefly, cortical tissues were mechanically dissociated using a Pasteur pipette and seeded into DMEM/high glucose (HyClone; Cytiva, SH30243.01) supplemented with 10% fetal bovine serum (FBS; Gibco; Thermo Fisher Scientific, Waltham, MA, USA), 10% horse serum (Gibco), and 1% penicillin–streptomycin (P/S, Gibco). Cells were maintained at 37 °C in a 5% CO₂ incubator. On day 3, the debris was removed by pipetting with PBS, and the medium was refreshed. When the cultures reached 80% confluence, cells were reseeded into 24-well plates at a density of 5 × 10^4^ cells/well. After 24 h, the medium was replaced with serum-free DMEM/high glucose and incubated for 16 h. Inflammation was induced by treatment with 100 ng/mL lipopolysaccharide (LPS; Millipore Sigma, Burlington, MA, USA) for 2 h, followed by treatment with 20% (v/v) cell-free supernatant (CFS) for 24 h.

#### Immunocytochemistry

Glial cells were fixed with 4% paraformaldehyde (Biosesang, Yongin, Korea) and immunostained with an anti-GFAP antibody conjugated to Cy3 (1:1000; Millipore Sigma) for astrocytes. Nuclei were counterstained with DAPI using the VectaShield antifade mounting medium (Vector Laboratories, Burlingame, CA, USA). Fluorescent images were acquired using a confocal microscope (LSM700; Zeiss, Oberkochen, Germany) or a slide scanner (Axio Scan.Z1; Zeiss).

#### Quantitative real-time PCR in glial cells

Primary astrocytes were seeded in 6-well plates at a density of 5 × 10^5^ cells/well. After 40 h, inflammation was induced by treatment with 100 ng/mL LPS for 2 h, followed by 20% (v/v) CFS for an additional 24 h. Total RNA was extracted using TRIzol reagent (Invitrogen; Thermo Fisher Scientific), and cDNA was synthesized using the TOPscript cDNA Synthesis Kit (Enzynomics, Daejeon, Korea). Quantitative PCR was performed using TOPreal SYBR Green qPCR PreMIX (Enzynomics) on a CFX96 Real-Time PCR System (Bio-Rad Laboratories, Hercules, CA, USA). Primer sequences used in this study are listed in Table S1, and RT-qPCR conditions are provided in Table S2. Gene expression levels were normalized to GAPDH and calculated using the 2^−ΔΔCt^ method (Livak and Schmittgen 2001). GAPDH was used as the reference gene because its Ct values showed limited variation across the experimental groups. Melting curve analysis was performed following amplification to confirm amplification specificity.

### SH-SY5Y cell experiments

#### SH-SY5Y cell culture and viability assay

SH-SY5Y human neuroblastoma cells (ATCC) were cultured in DMEM/F12 medium supplemented with 10% FBS (Corning, NY, USA) and 1% P/S. For viability assays, cells were seeded in 96-well plates at a density of 1 × 10^5^ cells/well and incubated for 24 h. The cells were then treated with CFS, LPS, CML (Millipore Sigma), or MGO (Millipore Sigma) for 24 h. After treatment, cells were washed twice with DPBS, and 200 μL of fresh medium and 20 μL of EZ-Cytox reagent (DoGenBio, Seoul, Korea) were added to each well. After 30 min of incubation, the absorbance was measured at 450 nm using a microplate reader (BioTek Epoch Microplate Spectrophotometer; Agilent Technologies, Santa Clara, CA, USA).

#### Intracellular ROS assay

Intracellular ROS levels were measured using 2′,7′-dichlorodihydrofluorescein diacetate (DCFH-DA; Millipore Sigma). SH-SY5Y cells were seeded in black 96-well plates at a density of 1 × 10^5^ cells/well, treated with CFS, LPS, or CML for 24 h, and then incubated with 25 μM DCFH-DA for 1 h at 37 °C in the dark. Fluorescence was measured at excitation and emission wavelengths of 485/535 nm. ROS levels were normalized to those of untreated controls and expressed as relative fluorescence units.

#### Quantitative real-time PCR in SH-SY5Y cells

SH-SY5Y cells were seeded in 6-well plates at a density of 1 × 10^6^ cells/well and incubated for 24 h. Cells were treated with 50% (v/v) CFS, LPS (1 μg/mL), or CML (10 μg/mL) for 24 h. Total RNA extraction, cDNA synthesis, and qPCR were performed as described above in the “Quantitative real-time PCR in glial cells” subsection. Primer sequences used in this study are listed in Table S3, and RT-qPCR conditions are provided in Table S4.

### Evaluation of probiotic properties under gastrointestinal conditions

#### Acid tolerance

LAB strains were cultured in MRS broth at 37 °C for 16 h and harvested by centrifugation (16,000 × g, 1 min). After washing with 0.88% NaCl, the cells were resuspended in 0.1 M glycine–HCl buffer (pH 2.5) and incubated at 37 °C for 2 h. After treatment, cells were washed, serially diluted, and plated on MRS agar. Viable colonies were counted after 20 h incubation at 37 °C. The survival rate (%) was calculated relative to untreated controls.

#### Bile and pancreatin tolerance

Each strain was inoculated (1%, v/v) into MRS broth containing 0.3% (w/v) oxgall or 0.5% (w/v) pancreatin (Millipore Sigma) and incubated at 37 °C for 24 h. Viable cell counts were determined by standard plate counting on MRS agar. Tolerance was expressed as the percentage of CFU compared to non-treated controls.

#### Auto-aggregation assay

The LAB strains were washed twice with PBS and adjusted to an OD₆₀₀ of 0.3. The suspension was incubated at 37 °C without disturbance, and OD₆₀₀ was measured at 0 h and 2 h using a microplate reader. Auto-aggregation (%) was calculated as the reduction in OD over time relative to the initial OD.

#### Adhesion to Caco-2 cells

Caco-2 cells (ATCC HTB-37) were cultured in DMEM supplemented with 10% FBS (Corning) and 1% P/S at 37 °C in a 5% CO₂ incubator. Cells (5 × 10^5^/well) were cultured in DMEM until reaching 80–90% confluence. LAB strains (5 × 10⁸ CFU) were added and incubated at 37 °C for 2 h. Nonadherent bacteria were removed by washing twice with DPBS. The cells were then treated with 0.25% trypsin–EDTA, followed by 0.1% Triton X-100 to release the adhered bacteria. The suspension was plated on MRS agar and incubated at 37 °C for 20 h. Adhesion was expressed as the percentage of viable adhered bacteria compared to the input.

#### Antioxidant capacity assay

The antioxidant capacity of *P. pentosaceus* TAP041 and TAP041 CFS was assessed using ABTS radical scavenging assays with slight modifications from previously reported methods (Jeong et al. 2023; Kim et al. 2021). *P. pentosaceus* TAP041 culture supernatant in MRS and CFS were mixed with pre-formed ABTS radical solution, incubated for 5 min at 37 °C. The reaction mixture was transferred to a 96-well plate for absorbance measurement at 734 nm. Radical-scavenging activity (%) was calculated as (1 − A_sample_/A_control_) × 100.

### Safety assessment of LAB strains

#### Antibiotic susceptibility

Each LAB strain was suspended in 0.88% NaCl and adjusted to a turbidity equivalent to 0.5 McFarland standard (1–5 × 10^8^ CFU/mL). The suspension was then spread evenly on MRS agar plates. ETEST strips (bioMérieux, Marcy-l’Étoile, France) for nine antibiotics were applied, and plates were incubated at 37 °C for 24 h. Minimum inhibitory concentrations (MICs) were determined and compared with EFSA (2012) breakpoint values. Strains with MIC values exceeding the cutoff were considered resistant.

#### Hemolytic activity

Hemolytic activity was assessed on blood agar base plates (Kisan Bio, Seoul, Korea) supplemented with 5% defibrinated sheep blood. Each strain was streaked on the plates and incubated anaerobically at 37 °C for 48 h. *Streptococcus pneumoniae* KCCM 41570 (α-hemolysis), *Streptococcus agalactiae* KCCM 40417 (γ-hemolysis), and *Streptococcus pyogenes* KCCM 40411 (β-hemolysis) were used as reference controls. Hemolytic activity was interpreted based on the presence of clear (β), greenish (α), or absent (γ) zones around colonies.

#### Cytotoxicity

Caco-2 cells were seeded into 96-well plates at a density of 5 × 10^4^ cells/well and incubated for 20 h. LAB strains were cultured in MRS broth at 37 °C for 18 h, harvested by centrifugation (20,781 × g, 1 min), washed three times with DPBS, and resuspended in DMEM. Caco-2 cells were exposed to bacterial suspensions at multiplicities of infection (MOI) of 125, 250, and 500 for 24 h. Cell viability was assessed using the EZ-Cytox reagent, with absorbance measured at 450 nm using a microplate reader. *Salmonella* Typhimurium ATCC 14028 was used as a positive control.

#### D-/L-lactic acid quantification

LAB strains were cultured in MRS broth at 37 °C for 24 h. The cultures were centrifuged at 16,000 × g for 10 min. The supernatants were used for quantification of D-/L-lactic acid using Enzytec Liquid D-/L-Lactic acid and D-Lactic acid kits (R-Biopharm AG, Darmstadt, Germany) according to the manufacturer’s protocol. The absorbance was measured at 340 nm using a microplate reader.

#### Bile salt hydrolase activity

Bile salt hydrolase (BSH) activity was evaluated by streaking strains on MRS agar supplemented with 0.5% (w/v) taurodeoxycholic acid (TDCA; Millipore Sigma) and incubating at 37 °C for 48 h. Formation of opaque precipitates around the colonies was interpreted as a positive BSH reaction. *Lacticaseibacillus plantarum* KU15122 was used as a positive control.

#### Toxic metabolite production

Urease activity was evaluated using Christensen urea agar base containing phenol red. Strains were streaked onto the medium and incubated anaerobically at 37 °C for 24 h. *Proteus mirabilis* KCCM 11381 was used as a positive control. A colour change from orange to pink was considered urease positive.

Indole production was assessed using tryptophan broth. After 18 h of incubation, five drops of Kovac’s reagent (Millipore Sigma) were added. Formation of a pink ring at the top of the medium was considered positive.

#### Biogenic amine production

Strains were cultured in MRS broth supplemented with 400 ppm each of arginine, histidine, lysine, ornithine, phenylalanine, and tryptophan, and 100 ppm of tyrosine (Millipore Sigma), and incubated at 37 °C for 24 h. The culture supernatant was derivatised and analysed by HPLC using a DIONEX UltiMate 3000 system (Thermo Fisher Scientific) with a UV detector (254 nm) and TC-C18 column (Agilent, 250 × 4.6 mm, 5 µm). Acetonitrile/water (55/45) was used as the mobile phase at a flow rate of 1 mL/min. Standards included agmatine, β-phenylethylamine, histamine, putrescine, serotonin, spermidine, tryptamine, and tyramine.

#### Enzymatic activity

The enzymatic activities of 19 hydrolases were determined using the API ZYM kit (bioMérieux, Marcy-l’Étoile, France). After 24 h cultivation in MRS broth at 37 °C, cells were harvested, washed, and resuspended in saline to 5–6 McFarland turbidity. A 65 µL aliquot of suspension was loaded into each well of the API ZYM strip and incubated at 37 °C for 4 h. ZYM A and B reagents were added, and enzymatic activity was recorded based on colour development.

### Whole-genome sequencing and functional annotation

The genomic DNA of *P. pentosaceus* TAP041 was extracted using a MagAttract HMW DNA Kit (Qiagen, Hilden, Germany). DNA quality was assessed by agarose gel electrophoresis, and concentration and purity were measured using a Qubit 2.0 fluorometer (Invitrogen, Thermo Fisher Scientific). For taxonomic identification, the 16S rRNA gene was amplified and sequenced using an ABI 3730 DNA Analyzer (Applied Biosystems, Thermo Fisher Scientific). Short-read libraries (~ 550 bp) were prepared using the TruSeq DNA Library LT Kit (Illumina, San Diego, CA, USA) and sequenced on a MiSeq platform (2 × 300 bp). Long-read sequencing was performed using the PacBio Sequel System (Pacific Biosciences, Menlo Park, CA, USA) with an SMRTbell library prepared from DNA fragments of > 3 kb. genome assembly and quality assessment were performed by CJ Bioscience (Seoul, Korea).

The hybrid genome assembly of *P. pentosaceus* TAP041 was performed using Unicycler v0.4.9 by integrating the Illumina and PacBio sequencing reads. Illumina reads were trimmed using Trimmomatic v0.36 and BBMap v38.32. Circular contigs were reoriented using Circlator v1.4.0. genome annotation was conducted with Prokka v1.14.6 (Seemann 2014). To determine the taxonomic classification of these strains, average nucleotide identity (ANI) values were calculated using the OrthoANI Tool (OAT), command-line version (Lee et al. 2016). genome-based digital DNA–DNA hybridization (dDDH) analysis was performed using the Type Strain Genome Server (TYGS, https://tygs.dsmz.de). Functional annotation of protein-coding sequences was carried out using eggNOG-mapper v2.1.12, with the eggNOG v5.0 database, and KEGG orthologs were assigned via BLASTKOALA (Kanehisa et al. 2016). Genes involved in glyoxalase detoxification, glutathione metabolism, antioxidant pathways, and biosynthesis of riboflavin, FMN, FAD, and folate were identified. carbohydrate-active enzymes (CAZymes) were annotated using the CAZy database. Antibiotic resistance genes were detected using ResFinder v4.6.0 (https://cge.food.dtu.dk/services/ResFinder) and Resistance Gene Identifier (RGI) v6.0.3 based on the CARD database v3.3.0 (https://card.mcmaster.ca). Virulence factors were predicted using VirulenceFinder v2.0 (https://cge.food.dtu.dk/services/VirulenceFinder). Horizontal gene transfer (HGT) events were predicted using HGTree2 (Kompramool et al. 2024), and pathogenic potential was assessed using PathogenFinder (https://cge.food.dtu.dk/services/PathogenFinder).

### Statistical analysis

All data are presented as mean ± standard deviation (SD) from three independent biological replicates (*n* = 3). Statistical analyses were performed using one-way analysis of variance, followed by Tukey’s post hoc test using GraphPad Prism (version 10.0, GraphPad Software, San Diego, CA, USA). Normality and homogeneity of variance were assessed using the Shapiro–Wilk and Levene’s tests, respectively, before one-way analysis of variance (ANOVA) followed by Tukey’s post hoc test. Differences were considered statistically significant at *p* < 0.05.

## Results

### Effects of LAB-derived cell-free supernatants on LPS-induced responses in primary glial cells

**Fig. 1:**
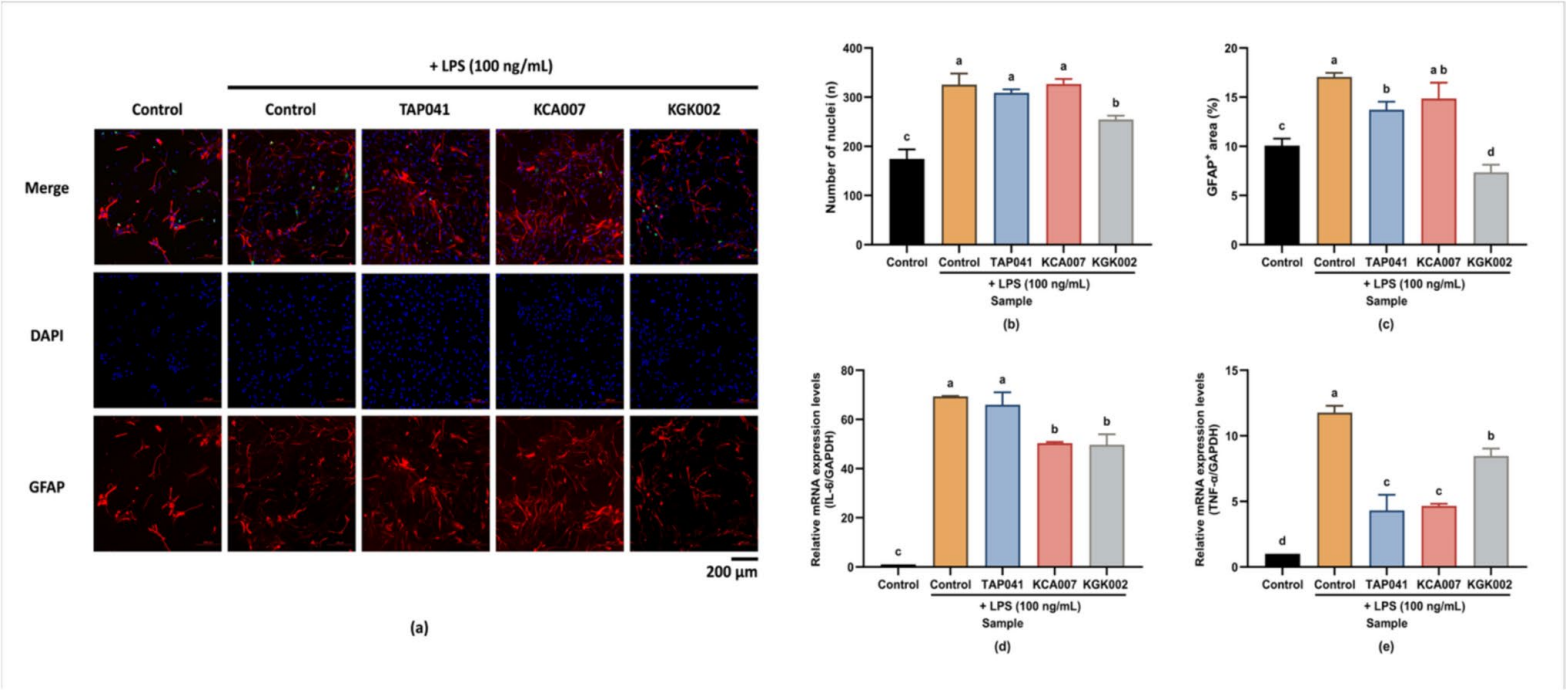
Effects of LAB-derived supernatants on LPS-induced glial activation in primary rat glial cells. (**a**) Representative immunofluorescence images of GFAP (red) and DAPI (blue) staining following treatment with LAB-derived cell-free supernatants (CFS, 20% v/v) under LPS (100 ng/mL) stimulation for 24 h. Scale bars: 200 µm. (**b**) Quantification of DAPI-positive nuclei. (**c**) GFAP-positive area (%). Relative mRNA expression levels of IL-6 (**d**) and TNF-α (**e**) were measured by RT-qPCR after 24 h CFS treatment. Data are expressed as mean ± SD (*n* = 3 independent biological experiments). Statistical analysis was performed using one-way ANOVA followed by Tukey’s post hoc test. Different letters indicate significant differences at *p* < 0.05

Primary rat glial cells were stimulated with LPS in the presence or absence of CFSs, and markers of glial activation were assessed. LPS stimulation significantly increased the number of DAPI-positive nuclei compared with untreated controls. Treatment with the CFS from *L. brevis* KGK002 significantly reduced this increase (Fig. 1b). GFAP immunostaining showed an increased GFAP-positive area in LPS-treated cells relative to untreated controls. Treatment with CFSs from *P. pentosaceus* TAP041 (TAP041 CFS) and *L. brevis* KGK002 significantly decreased the GFAP-positive area compared with the LPS control (Fig. 1c).

LPS significantly increased IL-6 and TNF-α mRNA expression in primary glial cells compared with untreated controls. Treatment with CFSs significantly reduced the expression of these cytokines relative to the LPS control (Fig. 1d–e). *P. pentosaceus* KCA007 and *L. brevis* KGK002 showed the largest reduction in IL-6 expression, whereas all three strains significantly reduced TNF-α expression compared with the LPS control, with *P. pentosaceus* TAP041 and KCA007 showing the most pronounced effects.

### Effects of LAB-derived cell-free supernatants on MGO-induced responses in SH-SY5Y cells

**Fig. 2:**
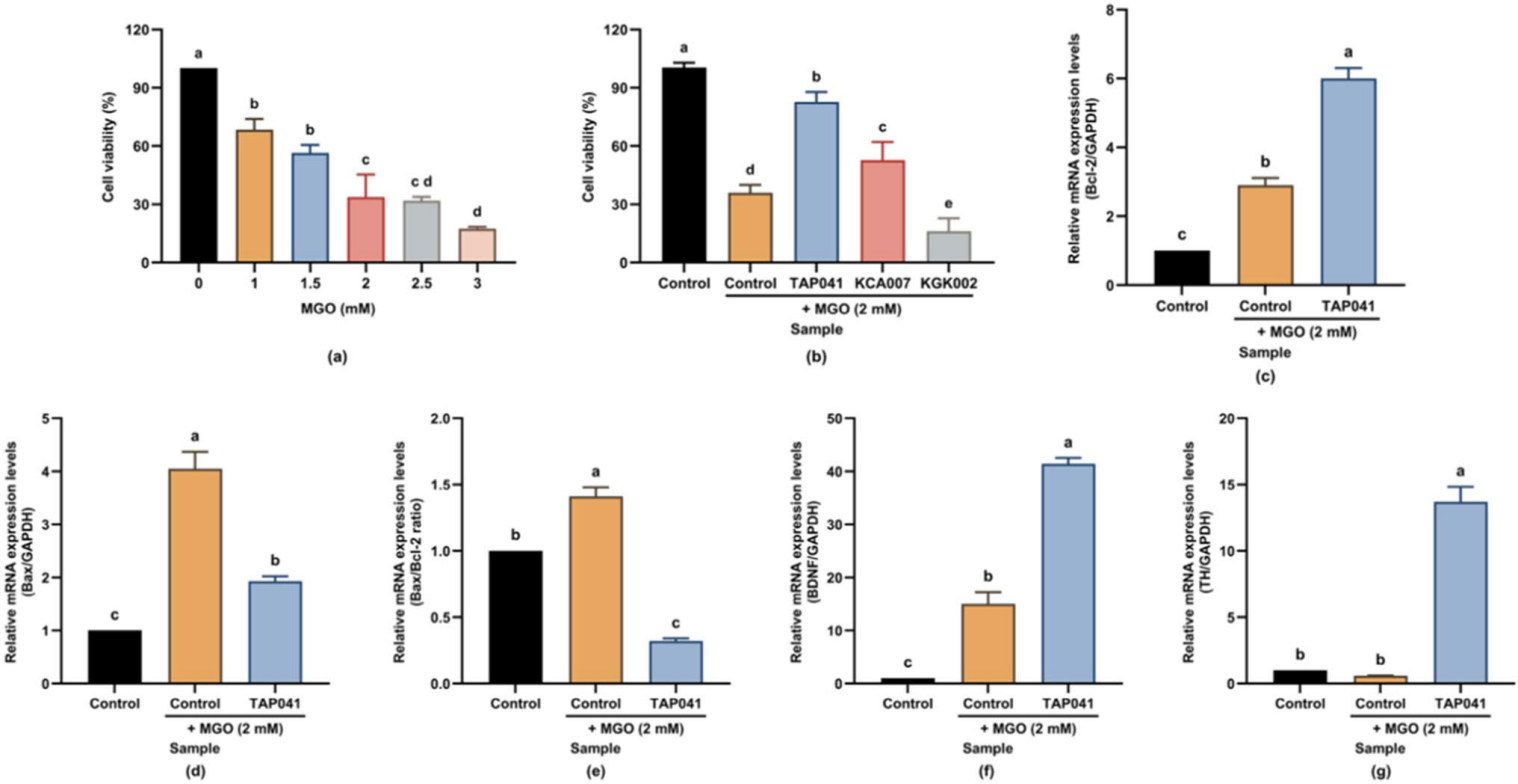
Protective effects of LAB-derived supernatants against MGO-induced stress in SH-SY5Y cells. (**a**) Cell viability following treatment with increasing concentrations of MGO (0–3 mM, 24 h). (**b**) Protective effects of LAB-derived cell-free supernatants (50% v/v, 24 h) under 2 mM MGO exposure. Relative mRNA expression levels of Bcl-2 (**c**), Bax (**d**), Bax/Bcl-2 ratio (**e**), BDNF (**f**), and TH (**g**) were assessed using RT-qPCR. Data are expressed as mean ± SD (*n* = 3 independent biological experiments). Statistical significance was determined by one-way ANOVA followed by Tukey’s post hoc test. Different letters indicate significant differences at *p* < 0.05

SH-SY5Y neuroblastoma cells were exposed to MGO in the presence or absence of CFSs. Cell viability decreased in a concentration-dependent manner following MGO treatment compared with untreated controls, and approximately 30% viability was observed at 2 mM MGO. This concentration was used for subsequent experiments (Fig. 2a). Exposure to 2 mM MGO significantly reduced cell viability compared with untreated controls. Treatment with CFSs significantly increased cell viability relative to the MGO control. Among the tested strains, *P. pentosaceus* TAP041 produced the largest increase in viability, followed by *P. pentosaceus* KCA007 (Fig. 2b).

MGO treatment significantly increased Bax and Bcl-2 mRNA expression, and elevated the Bax/Bcl-2 ratio compared with untreated controls (Fig. 2c–e). Treatment with *P. pentosaceus* TAP041 decreased Bax expression and increased Bcl-2 expression, resulting in a significantly lower Bax/Bcl-2 ratio compared with the MGO control. MGO treatment increased BDNF expression and had minimal effect on TH expression compared with the untreated control. Treatment with *P. pentosaceus* TAP041 significantly increased both BDNF and TH expression relative to the MGO control (Fig. 2f–g).

### Effects of cell-free supernatant from P. pentosaceus TAP041 on LPS- and CML-induced responses in SH-SY5Y cells

**Fig. 3:**
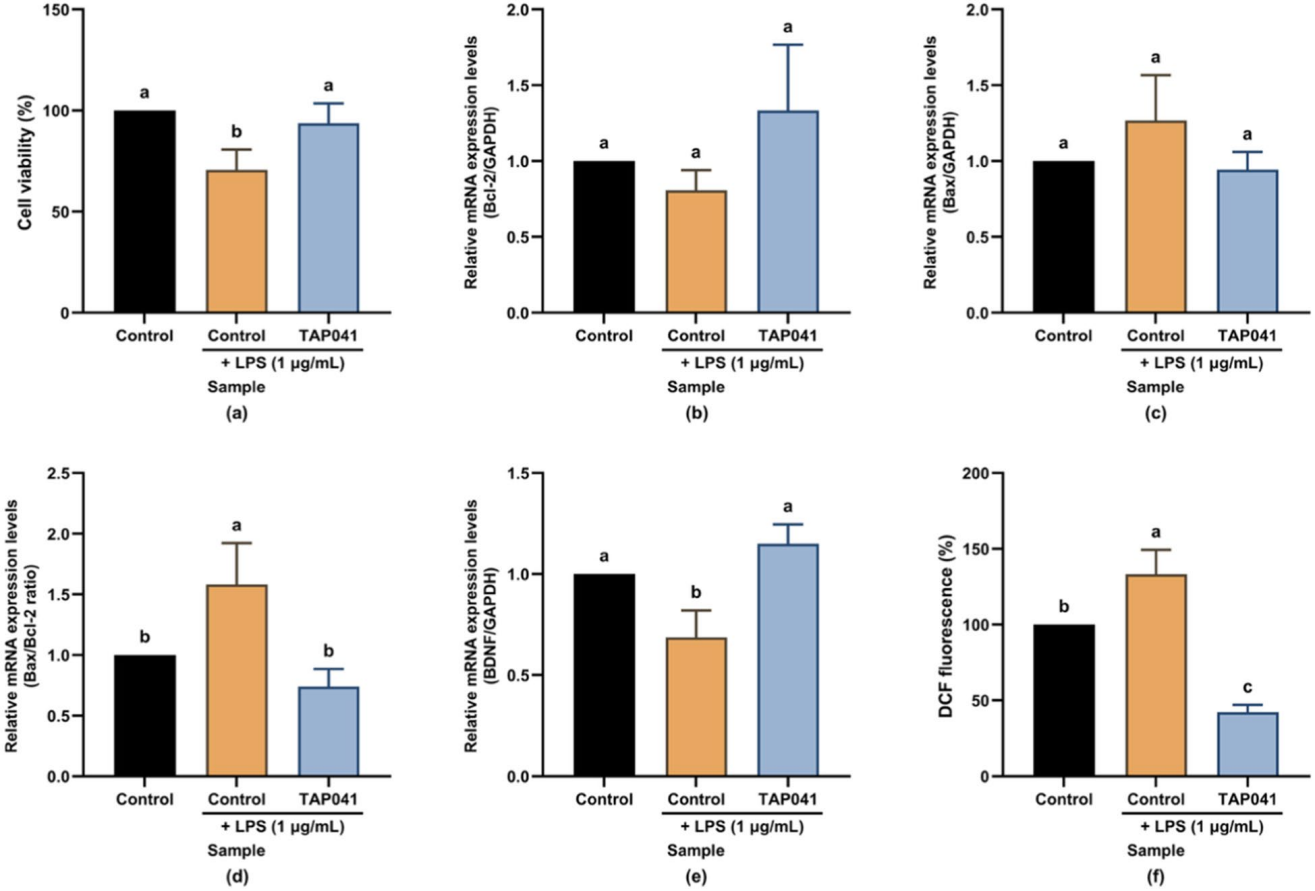
Protective effects of cell-free supernatant from *P. pentosaceus* TAP041 (TAP041 CFS) against LPS-induced stress in SH-SY5Y cells. (**a**) cell viability following co-treatment with TAP041 CFS (50% v/v) and LPS (1 µg/mL) for 24 h. Relative mRNA expression levels of Bcl-2 (**b**), Bax (**c**), Bax/Bcl-2 ratio (**d**), and BDNF (**e**) were assessed by RT-qPCR. **(f) **intracellular ROS levels measured by DCFH-DA staining. Data are expressed as mean ± SD (*n* = 3 independent biological experiments). Statistical significance was determined by one-way ANOVA followed by Tukey’s post hoc test. Different letters indicate significant differences at *p* < 0.05

SH-SY5Y neuroblastoma cells were treated with LPS (1 μg/mL) or CML in the presence or absence of TAP041 CFS. LPS exposure significantly reduced cell viability compared with untreated controls. Co-treatment with TAP041 CFS significantly increased cell viability relative to the LPS control (Fig. 3a).

Bax and Bcl-2 mRNA expression did not significantly differ among groups (Fig. 3b–c). However, LPS treatment significantly increased the Bax/Bcl-2 ratio compared with untreated controls, whereas TAP041 CFS treatment significantly decreased this ratio relative to the LPS control (Fig. 3d). LPS treatment decreased BDNF expression compared with untreated controls, whereas TAP041 CFS treatment significantly increased BDNF expression relative to the LPS control (Fig. 3e). LPS treatment increased intracellular ROS levels compared with untreated controls, and TAP041 CFS treatment significantly reduced ROS levels compared with the LPS control (Fig. 3f).

**Fig. 4:**
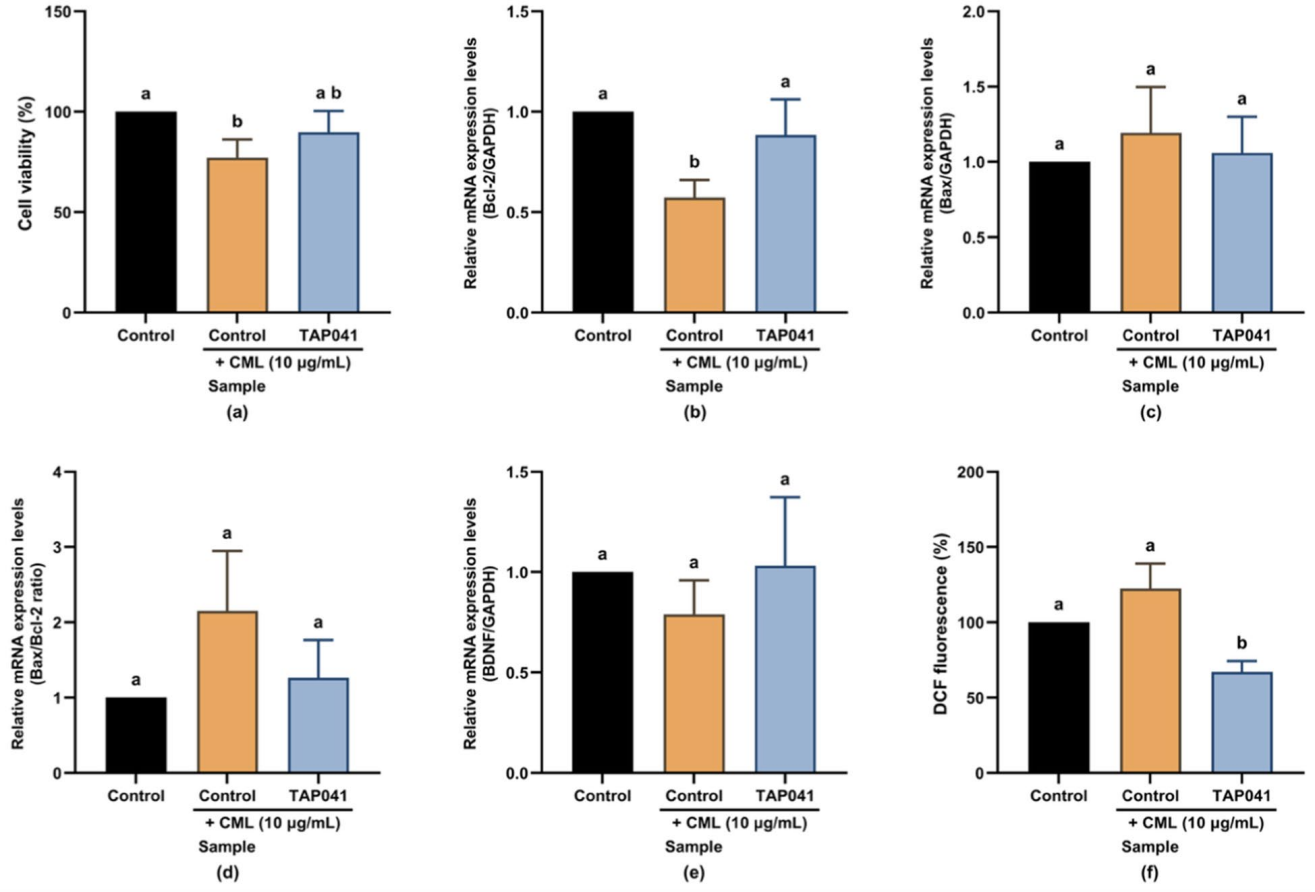
Protective effects of cell-free supernatant from *P. pentosaceus* TAP041 (TAP041 CFS) against CML-induced stress in SH-SY5Y cells. (**a**) Cell viability following co-treatment with TAP041 CFS (50% v/v) and CML (10 µg/mL) for 24 h. Relative mRNA expression levels of Bcl-2 (**b**), Bax (**c**), Bax/Bcl-2 ratio (**d**), and BDNF (**e**) were assessed by RT-qPCR. (**f)** Intracellular ROS levels measured by DCFH-DA staining. Data are expressed as mean ± SD (*n* = 3 independent biological experiments). Statistical significance was determined by one-way ANOVA followed by Tukey’s post hoc test. Different letters indicate significant differences at *p* < 0.05

CML exposure significantly reduced cell viability compared with untreated controls; however, co-treatment with TAP041 CFS did not produce a statistically significant recovery in cell viability relative to the CML control (Fig. 4a). Among apoptosis-related markers, CML treatment significantly decreased Bcl-2 expression compared with untreated controls, whereas TAP041 CFS treatment significantly increased Bcl-2 expression relative to the CML control. Bax expression and the Bax/Bcl-2 ratio did not significantly differ among groups (Fig. 4b–d). BDNF expression was also not significantly altered by CML treatment or TAP041 CFS co-treatment (Fig. 4e). CML treatment did not markedly elevate intracellular ROS levels under the present experimental conditions; however, TAP041 CFS treatment significantly reduced ROS levels compared with the CML control (Fig. 4f).

### Probiotic characteristics of LAB strains under simulated gastrointestinal conditions

**Fig. 5:**
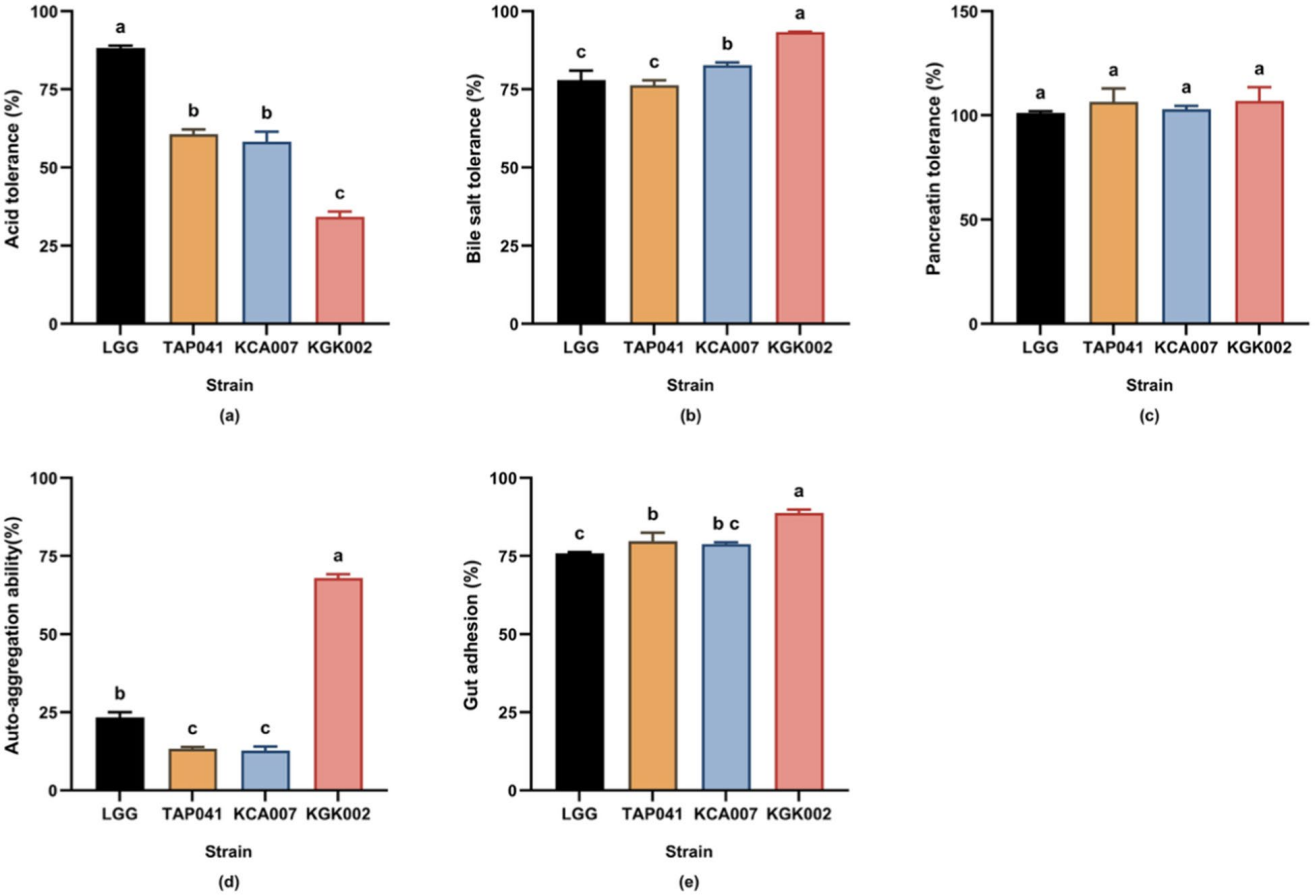
Probiotic properties of LAB strains: Acid tolerance (0.1 M glycine–HCl, pH 2.5, 2 h) (**a**), bile salt tolerance (0.3% w/v oxgall, 24 h) (**b**), pancreatin tolerance (0.5% w/v pancreatin, 24 h) (**c**), auto-aggregation ability (2 h) (**d**), and adhesion to Caco-2 intestinal epithelial cells (**e**). The probiotic potential of *Lacticaseibacillus rhamnosus* GG (LGG), *Pediococcus pentosaceus* TAP041 and KCA007, and *Levilactobacillus brevis* KGK002 was evaluated under simulated gastrointestinal conditions. Data are expressed as mean ± SD (*n* = 3 independent biological experiments). Statistical significance was determined by one-way ANOVA followed by Tukey’s post hoc test. Different letters indicate significant differences at *p* < 0.05

The probiotic-related properties of LAB strains, including acid tolerance, bile salt tolerance, pancreatin resistance, auto-aggregation, and adhesion to intestinal epithelial cells, were evaluated under simulated gastrointestinal conditions and compared with those of *Lacticaseibacillus rhamnosus* GG (LGG) (Fig. 5). LGG showed the highest survival under acidic conditions, whereas *L. brevis* KGK002 showed the lowest survival. *P. pentosaceus* TAP041 and KCA007 exhibited intermediate survival, with approximately 60% viability (Fig. 5a). Under bile salt conditions, *L. brevis* KGK002 showed the highest viability, and other strains, including LGG and *P. pentosaceus* TAP041, also maintained survival (Fig. 5b). No significant differences in pancreatin tolerance were observed among the strains (Fig. 5c). Surface property analysis showed that *L. brevis* KGK002 exhibited the highest auto-aggregation and adhesion to Caco-2 cells (Fig. 5d–e), and all tested strains adhered to Caco-2 cells. In the ABTS radical scavenging assay, TAP041 CFS exhibited an antioxidant activity of 89.72 ± 3.53%, whereas the MRS culture supernatant showed 55.65 ± 0.17%. Notably, the activity of TAP041 CFS remained lower than that of the 2 mM ascorbic acid control (124.15 ± 1.50%).

### Safety-related characteristics of LAB strains

Antibiotic susceptibility, haemolytic activity, cytotoxicity, metabolic indicators, biogenic amine production, and enzymatic activity were evaluated to assess safety-related characteristics of the LAB strains. All tested strains showed MIC values below EFSA cut-off thresholds for ampicillin, gentamicin, kanamycin, erythromycin, clindamycin, tetracycline, and chloramphenicol, whereas vancomycin resistance was observed in all strains (Table S5). All strains exhibited γ-haemolysis on blood agar and showed no reduction in Caco-2 cell viability (Fig. S1a–b). Low levels of D-lactic acid were detected in all strains (Fig. S1c), and bile salt hydrolase, urease, and tryptophanase activities were not detected (Fig. S1d–f). Histamine and tyramine were not detected, whereas low levels of tryptamine were observed in *P. pentosaceus* KCA007 and serotonin in *L. brevis* KGK002 (Table S6). Enzymatic profiling showed the absence of β-glucuronidase activity and the presence of β-galactosidase activity in all strains (Table S7).

### Genomic features of P. pentosaceus TAP041

**Fig. 6:**
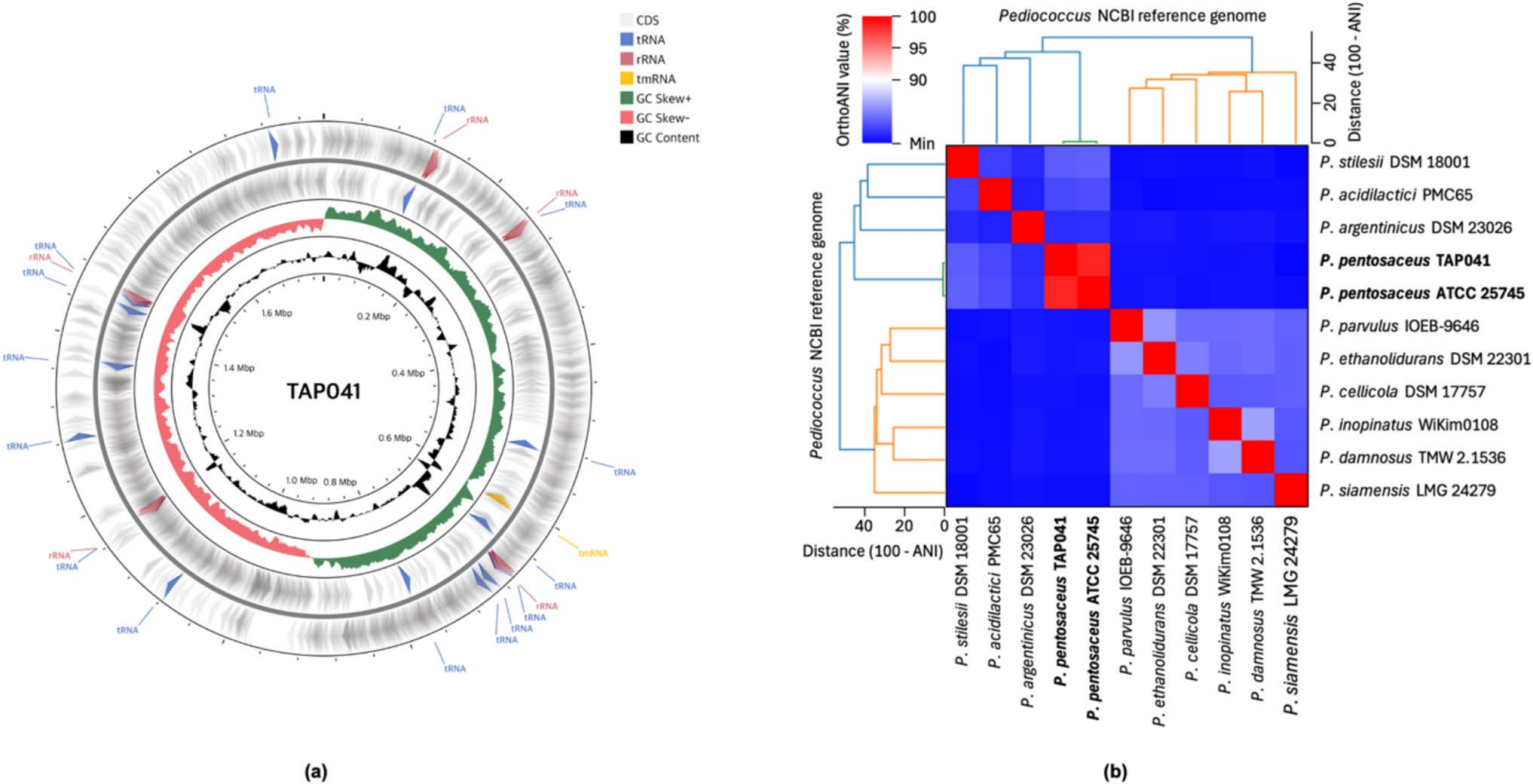
Circular genome map and average nucleotide identity (ANI) analysis of *Pediococcus pentosaceus* TAP041. (**a)** circular genome map showing Prokka-annotated coding sequences (CDSs), tRNAs, rRNAs, and tmRNA, along with GC content and GC skew (positive in green, negative in pink). The total genome size is 1,785,489 bp with a GC content of 37.2%. (**b)** Heatmap of pairwise OrthoANI values among *Pediococcus* type strains. Colour scale represents OrthoANI values (%) with darker colours indicating higher identity

The complete genome of *P. pentosaceus* TAP041 was assembled into a single circular contig of 1,785,489 bp with a GC content of 37.2% (Table 1). The genome contained 1721 predicted coding sequences (CDSs), 15 rRNA genes, and 55 tRNA genes. genome annotation identified CDSs, rRNAs, tRNAs, and tmRNAs across the chromosome, together with GC content and GC skew profiles (Fig. 6a). Average nucleotide identity (ANI) analysis showed > 98% identity between *P. pentosaceus* TAP041 and reference *P. pentosaceus* strains, including ATCC 25745 (Fig. 6b). Digital DNA-DNA hybridization (dDDH) values between *P. pentosaceus* TAP041 and *P. pentosaceus* ATCC 25745 were 81.1% (d0), 88.6% (d4), and 85.3% (d6).

**Table 1:** Genome features of *Pediococcus pentosaceus* TAP041

Strain	Number of contigs	Genome size (bp)	GC content (%)	Number of CDSs	Number of rRNA genes	Number of tRNA genes
TAP041	1	1,785,489	37.2	1721	15	55

### Functional genome annotation of P. pentosaceus TAP041

**Fig. 7:**
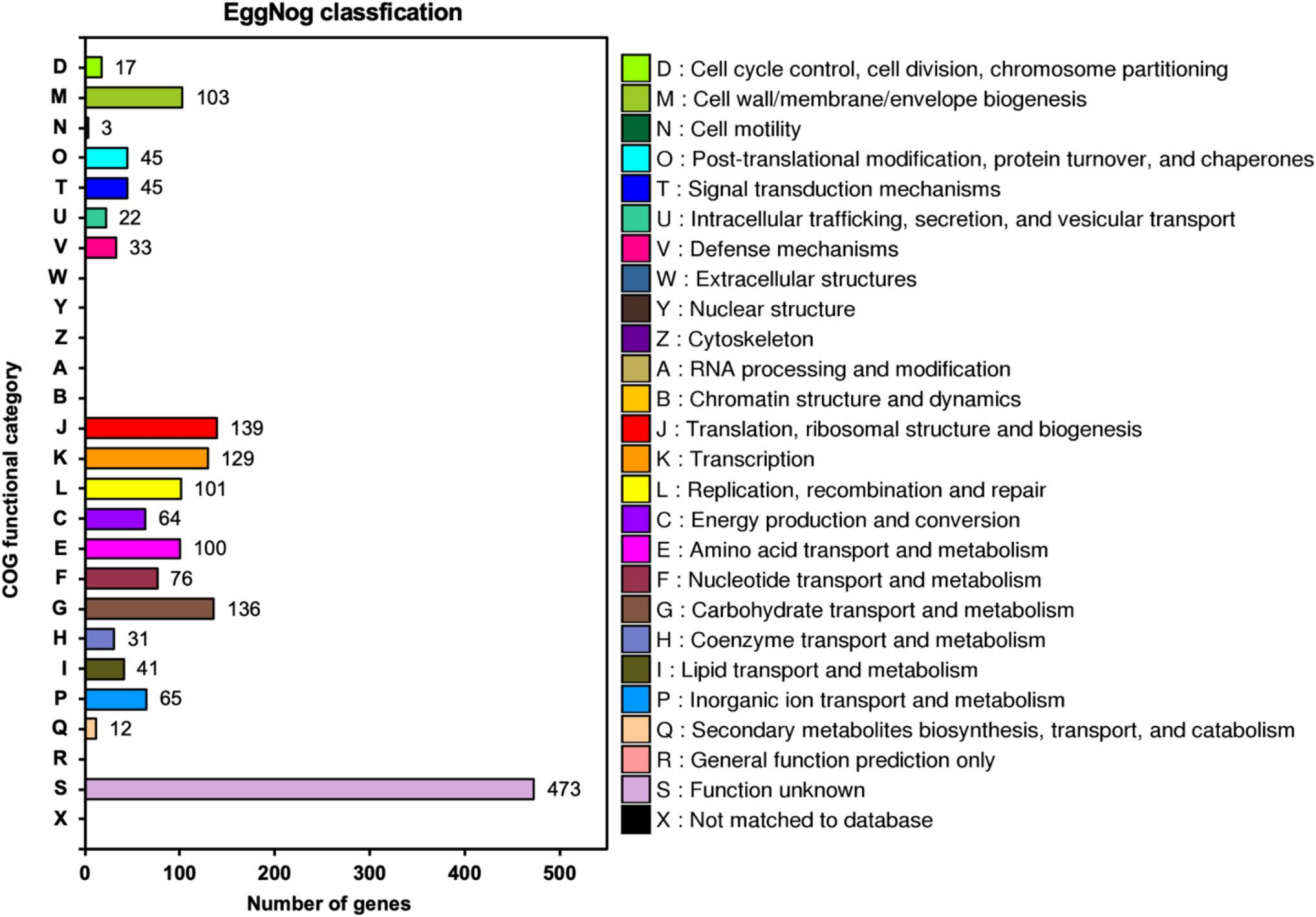
eggNOG functional annotation of *P. pentosaceus* TAP041. Distribution of 1635 protein-coding sequences across COG functional categories. The *X*-axis indicates the number of genes assigned to each COG functional category, and the *Y*-axis shows category abbreviations (D–X). Detailed category descriptions are provided in the figure legend

**Fig. 8:**
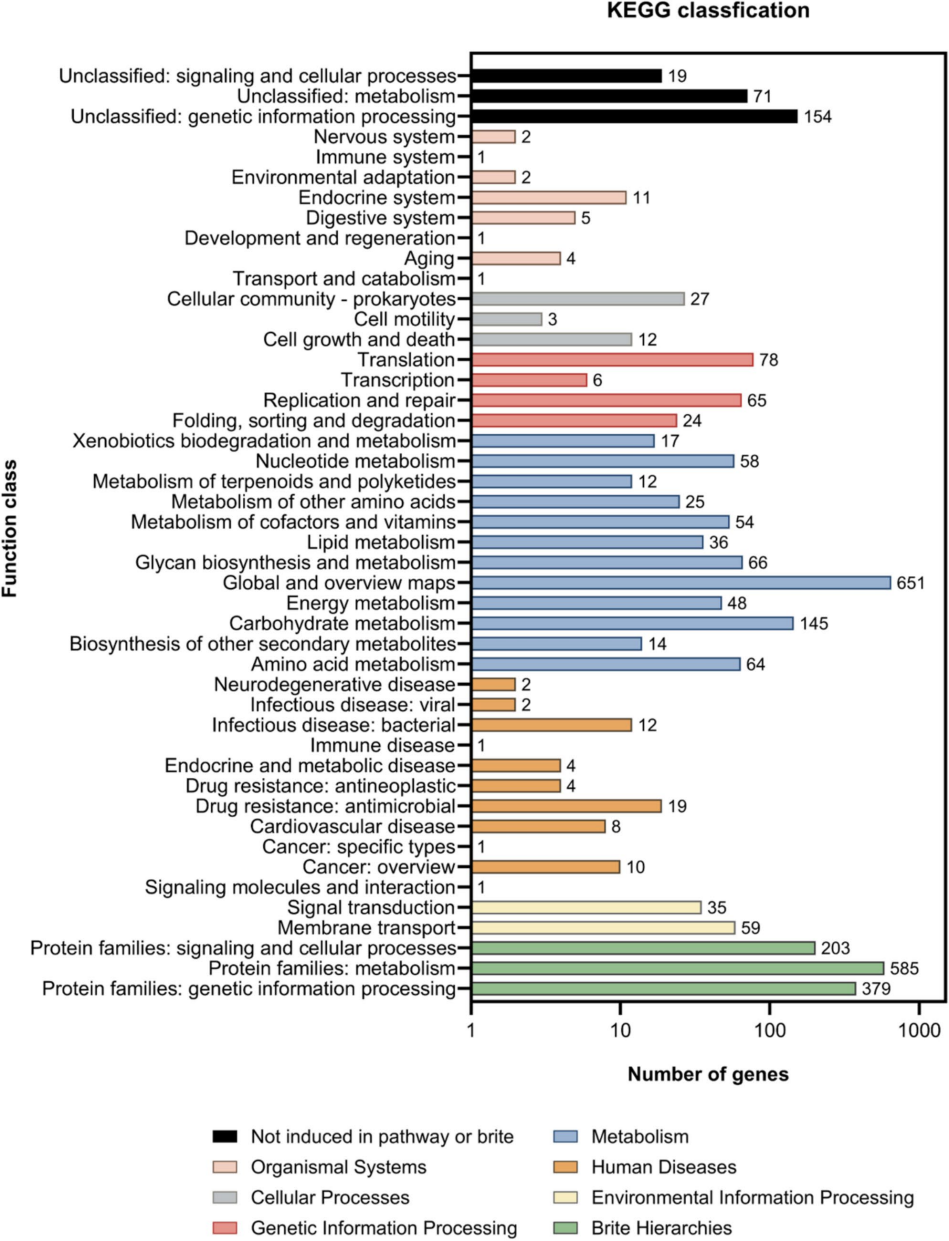
KEGG functional annotation of *P. pentosaceus* TAP041. Distribution of 1,044 KEGG-annotated genes across functional pathway categories. The *X*-axis (log scale) indicates the number of genes per category, and the *Y*-axis shows the function class. Colour codes correspond to the major KEGG groups (Metabolism, Genetic Information Processing, Cellular Processes, Organismal Systems, Human Diseases, Brite Hierarchies, Environmental Information Processing)

The genome of *P. pentosaceus* TAP041 was functionally annotated using the eggNOG, KEGG, and dbCAN databases (Table S8). Of the 1721 predicted coding sequences (CDSs), 1635 were assigned to functional categories based on eggNOG classification. The most represented categories were translation, ribosomal structure and biogenesis (J; *n* = 139), carbohydrate transport and metabolism (G; *n* = 136), and transcription (K; *n* = 129) (Fig. 7). KEGG pathway mapping assigned 1044 genes (60.7% of CDSs) to functional pathways. The most represented categories included protein families for genetic information processing (*n* = 166), genetic information processing pathways such as replication, transcription, and translation (*n* = 154), and carbohydrate metabolism (*n* = 121) (Fig. 8). Carbohydrate-active enzyme analysis identified 95 genes, including 37 glycoside hydrolases (GHs), 38 glycosyltransferases (GTs), 5 carbohydrate esterases (CEs), and 7 carbohydrate-binding modules (CBMs).

### Genome-encoded antioxidant, glyoxalase, and B-vitamin biosynthesis genes

Genome annotation of *P. pentosaceus* TAP041 identified genes associated with oxidative stress response and dicarbonyl detoxification. The genome contained glyoxalase system genes, including lactoylglutathione lyase (glyoxalase I) and D-lactate dehydratase (glyoxalase III). Genes involved in glutathione metabolism, such as glutathione disulfide reductase (glr) and hydroxyacylglutathione hydrolase (gloB), were also detected. Additionally, genes encoding antioxidant enzymes, including NADH peroxidase and thiol peroxiredoxin, and a peroxide-responsive transcriptional regulator (PerR) were annotated, indicating the presence of enzymatic systems for peroxide detoxification, redox balance maintenance, and reactive carbonyl compound metabolism.

Annotation of B-vitamin biosynthesis genes revealed riboflavin (vitamin B₂) pathway genes, including riboflavin synthase, riboflavin kinase, and genes for FMN/FAD metabolism, as well as a predicted riboflavin transporter (FmnP). Folate (vitamin B₉)-related genes were also identified, including 5-formyltetrahydrofolate cyclo-ligase, dihydrofolate reductase, dihydrofolate synthase, formate-tetrahydrofolate ligase, methylenetetrahydrofolate dehydrogenase (NADP⁺), and a folate transporter (FolT), indicating potential capacity for B-vitamin biosynthesis and transport.

### Genome-based safety assessment

Genome analysis of *P. pentosaceus* TAP041 revealed no acquired antibiotic resistance genes. ResFinder did not detect known resistance determinants, and CARD analysis identified only one strict hit and 104 loose hits, none of which indicated clinically relevant transferable resistance. A vanT-like gene shared 32% identity with the reference vanT gene, and a loose vatB hit showed 64% identity, suggesting these are distant homologs rather than functional resistance genes. KEGG annotation identified intrinsic resistance mechanisms typical of LAB, including an lsa-family ABC transporter, a putative penP β-lactamase homolog, the dltABCD operon, and an abcA (bmrA) multidrug efflux pump. Horizontal gene transfer analysis predicted 14 HGT-related genes (0.81% of the genome), none linked to antibiotic resistance, and no plasmids were detected. These results indicate the absence of transmissible resistance genes and the presence of only intrinsic resistance traits, consistent with probiotic safety.

Virulence-associated genes were not detected by VirulenceFinder. PathogenFinder predicted a very low probability of human pathogenicity (16.3%), and no matches to known pathogenic families were observed, confirming the strain’s non-pathogenic character. Collectively, these genomic findings support the safety of *P. pentosaceus* TAP041 for probiotic applications, with no identified traits that could compromise food safety or human health.

## Discussion

Neurodegenerative disorders are increasingly recognised as multifactorial conditions in which oxidative, inflammatory, and glycation-associated stress pathways converge to drive neuronal dysfunction. In the present study, in vitro cellular models were used to evaluate whether metabolites secreted by *P. pentosaceus* TAP041 modulate stress responses relevant to neurodegeneration. TAP041 CFS reduced intracellular ROS accumulation, attenuated pro-inflammatory cytokine expression in primary glial cells, and favorably modulated transcriptional markers of apoptosis and neuronal function in SH-SY5Y cells under MGO-, LPS-, and CML-induced stress conditions. Notably, while many previous studies of probiotic-associated neuroprotection have focused on individual stress modalities, the present findings suggest that secreted metabolites from *P. pentosaceus* TAP041 may concurrently influence oxidative, inflammatory, and apoptosis-related responses under the tested conditions. This pattern is consistent with coordinated modulation across multiple cellular stress axes, although the underlying signaling mechanisms were not directly examined, and mechanistic conclusions therefore remain tentative. genome analysis identified predicted pathways associated with antioxidant defense, glyoxalase-related detoxification, and B-vitamin biosynthesis, which are consistent with but do not directly establish the observed phenotypes. Supporting the genome-based predictions at the biochemical level, TAP041 CFS exhibited significantly higher ABTS radical scavenging activity than control, providing direct evidence that secreted antioxidant constituents are present in the CFS. Collectively, the present findings are consistent with a postbiotic-like mode of action (Salminen et al. 2021), in which secreted bacterial metabolites exert cytoprotective activity at the cellular level, without implying formal postbiotic classification. These observations provide a basis for future studies examining the mechanistic and physiological relevance of *P. pentosaceus* TAP041-derived metabolites in the context of AGE- and inflammation-associated neurodegeneration.

Attenuation of LPS-induced GFAP immunoreactivity and pro-inflammatory cytokine expression in primary glial cells suggests that metabolites secreted by *P. pentosaceus* TAP041 are capable of modulating astrocytic reactivity under acute inflammatory conditions. Reduced GFAP-positive area following TAP041 CFS treatment indicates suppression of reactive astrogliosis, a hallmark of neuroinflammation that contributes to neuronal damage in multiple neurodegenerative conditions. Concomitant reductions in IL-6 and TNF-α mRNA expression further support an anti-inflammatory effect at the transcriptional level, with *P. pentosaceus* TAP041 and KCA007 showing the most pronounced suppression of TNF-α among the tested strains. These observations are consistent with previous reports of reduced glial activation and inflammatory mediator expression following probiotic-related interventions in animal models of neurodegeneration (Aytekin Sahin et al. 2022; Zhang et al. 2024). In particular, suppression of IL-6 and TNF-α through inhibition of the TLR4/NF-κB signalling axis has been described in senescence-accelerated mouse models receiving probiotic supplementation (Yang et al. 2020). Whereas those studies employed live bacterial administration, the present findings indicate that secreted metabolites alone may be sufficient to attenuate LPS-induced glial inflammatory responses under the tested in vitro conditions. Nevertheless, the specific intracellular signalling pathways mediating these effects were not directly investigated in the present study, and therefore mechanistic interpretation remains tentative.

Under MGO-induced stress, SH-SY5Y cell viability was markedly reduced, and the Bax/Bcl-2 mRNA ratio was significantly elevated, consistent with activation of the intrinsic apoptotic pathway. Of note, both Bax and Bcl-2 expression levels were simultaneously increased following MGO treatment, a pattern that has been interpreted as a compensatory pro-survival response under oxidative stress conditions (Chetsawang et al. 2006). TAP041 CFS treatment was associated with restored cell viability and a reduced Bax/Bcl-2 ratio, achieved through coordinated downregulation of Bax and upregulation of Bcl-2. This pattern is suggestive of a partial re-establishment of apoptotic balance rather than complete suppression of apoptosis-related transcription, and should be interpreted at the mRNA level in the absence of protein-level confirmation. In addition, TAP041 CFS treatment was associated with increased BDNF and TH mRNA expression relative to the MGO control, markers linked to neuronal survival and dopaminergic function, respectively. Comparable modulation of BDNF, TH, and the Bax/Bcl-2 ratio by LAB-derived secreted factors has been reported for *Lactiplantibacillus plantarum* and *P. pentosaceus* strains in SH-SY5Y cells subjected to oxidative stress, with activation of the Nrf2/Keap1/HO-1 axis proposed as a contributing upstream mechanism (Bock et al. 2023; Cheon et al. 2021; Lee et al. 2025). Although Nrf2 signaling was not directly examined in the present study, the observed transcriptional response pattern is consistent with those earlier findings. Collectively, these results indicate that TAP041 CFS modulates apoptosis-related gene expression and neurotrophic marker transcription under MGO-induced stress conditions, though the functional significance of these transcriptional changes requires further validation at the protein level.

The observed cellular responses under MGO-induced stress may also be related to the carbonyl-detoxifying capacity of *P. pentosaceus* TAP041, a property that has been less commonly characterized in previous probiotic neuroprotection studies. Prior work from our group demonstrated that *P. pentosaceus* TAP041 reduces MGO and glyoxal (GO) concentrations under co-incubation conditions, with MGO reduction reaching 94.4% at 523 ppm and GO reduction reaching 31.4% at 95.2 ppm, both exceeding the activity of the reference strain *Lactococcus lactis* KF140 (Lee and Park 2024). This strain-level carbonyl-detoxifying activity provides functional context for interpreting the cellular protection observed in the present MGO model, and is further supported by the identification of glyoxalase system genes in the *P. pentosaceus* TAP041 genome, including lactoylglutathione lyase (glyoxalase I), hydroxyacylglutathione hydrolase (gloB), and D-lactate dehydratase (glyoxalase III). Given the established role of MGO accumulation in glycation-mediated neuronal injury and apoptosis (Frandsen and Narayanasamy 2017), this genomic repertoire may contribute to reducing the intracellular carbonyl burden and, consequently, to attenuating downstream stress responses. The capacity of lactic acid bacteria to metabolize reactive dicarbonyl compounds has also been reported more broadly, including thiol-independent MGO metabolism in species formerly classified under *Lactobacillus* sensu lato and since reclassified into multiple genera (Gandhi et al. 2018; Zheng et al. 2020). Together, the prior functional data, the genome-encoded glyoxalase pathway, and the CFS-level antioxidant activity demonstrated in the present study collectively provide converging, though associative, evidence for a potential capacity for carbonyl stress handling in *P. pentosaceus* TAP041. Nevertheless, direct measurement of glyoxalase enzyme activity in the CFS or within the neuronal experimental system was not performed, and causal attribution therefore requires further investigation.

The magnitude and pattern of cellular protection afforded by TAP041 CFS differed between the LPS- and CML-induced stress conditions, suggesting that the extent of cytoprotective activity may vary depending on the nature and intensity of the stressor. Under LPS stimulation, TAP041 CFS significantly reduced intracellular ROS accumulation, normalized the Bax/Bcl-2 ratio, and restored BDNF expression in SH-SY5Y cells, indicative of coordinated modulation of oxidative and apoptosis-related transcriptional responses. These findings are consistent with previous reports demonstrating that probiotic-related interventions attenuate LPS-induced neurotoxicity through antioxidant activity and suppression of inflammatory signalling (Frank et al. 2019; Zhu et al. 2024). In contrast, the effects observed under CML exposure were comparatively limited. Although CML treatment significantly reduced cell viability, co-treatment with TAP041 CFS did not produce statistically significant recovery in viability, and neither the Bax/Bcl-2 ratio nor BDNF expression was significantly altered relative to the CML control. Of note, CML did not markedly elevate intracellular ROS levels under the present experimental conditions; however, TAP041 CFS treatment was associated with a significant reduction in basal ROS accumulation relative to the CML control, suggesting that antioxidant activity of the CFS may be retained even in the absence of pronounced oxidative stress induction. The limited overall protective response under CML may reflect the distinct mechanism by which CML exerts cellular toxicity, primarily through AGE–RAGE receptor-mediated signalling and associated oxidative injury (Haddad et al. 2019; Takeda et al. 2001), rather than through the acute ROS burst characteristic of LPS stimulation. In the absence of RAGE pathway analysis, the mechanistic basis for the differential response between stress conditions could not be fully resolved in the present study. Additionally, while β-galactosidase activity was detected in all tested strains, and β-galactosidase-producing bacteria have been reported to reduce dietary CML absorption (Park et al. 2022), whether enzymatic modulation of CML contributes to the observed cellular responses under the present experimental conditions was not evaluated. Collectively, these findings suggest that TAP041 CFS exerts more pronounced protective effects under acute inflammatory oxidative stress than under the milder or mechanistically distinct AGE-related conditions tested here, and underscore the importance of stress model selection when evaluating the cytoprotective potential of probiotic-derived metabolites.

Survival under simulated gastrointestinal conditions is a prerequisite for probiotic functionality, as strains must withstand the low pH of the stomach and the bile salt concentrations encountered in the small intestine to reach the colon in sufficient numbers. All tested LAB strains maintained substantial viability following exposure to acidic conditions (pH 2.5), bile salts, and pancreatic enzymes, indicating a capacity to tolerate transient gastrointestinal stress, although in vivo persistence and colonisation were not directly assessed in the present study. Among the strains evaluated, LGG exhibited the highest survival under acidic conditions, whereas *P. pentosaceus* TAP041 and KCA007 maintained approximately 60% viability under the same conditions, reflecting intermediate but functionally relevant acid tolerance. Under bile salt challenge, *L. brevis* KGK002 exhibited the highest viability, while *P. pentosaceus* TAP041 and LGG also maintained substantial survival. No significant differences in pancreatin tolerance were observed among strains. Adhesion to intestinal epithelial cells and auto-aggregation capacity are considered supportive traits for transient mucosal colonisation and host-microbe interaction. Among the tested strains, *L. brevis* KGK002 exhibited the highest auto-aggregation ability and adhesion to Caco-2 cells; however, this strain also showed comparatively lower acid resistance, indicating a trade-off between surface properties and gastric stress tolerance. In contrast, *P. pentosaceus* TAP041 demonstrated balanced performance across the evaluated probiotic-related criteria, maintaining both gastrointestinal stress tolerance and intestinal adhesion capacity. Taken together, these characteristics are consistent with established criteria for the in vitro assessment of probiotic potential, though long-term colonisation capacity and clinical efficacy in the context of gut-brain axis modulation remain to be evaluated in appropriate in vivo models.

Comprehensive safety assessment is a fundamental prerequisite for probiotic development. Phenotypic antibiotic susceptibility testing indicated that all tested strains were susceptible to clinically relevant antibiotics within EFSA-defined breakpoint values. The exception was vancomycin resistance, which is well recognized as an intrinsic and non-transferable trait in *Pediococcus* spp. and related lactic acid bacteria, and is therefore not considered a safety concern in this context (Gueimonde et al. 2013; Singla et al. 2018). genome-level analysis further corroborated these phenotypic findings: no acquired or transferable antibiotic resistance determinants were identified by ResFinder, and CARD analysis revealed no clinically relevant resistance genes. The vanT-like and vatB homologs detected showed low sequence identity to their respective reference genes (32% and 64%, respectively), consistent with distant homologs rather than functional resistance determinants. Intrinsic resistance-associated genes identified by KEGG annotation, including an lsa-family ABC transporter, a putative penP β-lactamase homolog, the dltABCD operon, and an abcA multidrug efflux pump, are characteristic of lactic acid bacteria and were not associated with mobile genetic elements. The low proportion of predicted horizontal gene transfer events (14 genes, 0.81% of the genome) and the absence of plasmid sequences further reduce the likelihood of resistance dissemination, supporting a chromosomally stable and transferable-resistance-free genomic architecture. Beyond antibiotic resistance, all strains exhibited γ-hemolysis and showed no reduction in Caco-2 cell viability, indicating non-hemolytic and non-cytotoxic phenotypes. No known virulence factors were detected by VirulenceFinder, and PathogenFinder predicted a low probability of human pathogenicity (16.3%), with no matches to known pathogenic families, collectively supporting the non-pathogenic character of *P. pentosaceus* TAP041. Metabolic safety profiling demonstrated low D-lactic acid production and absence of β-glucuronidase activity in all strains, both of which are considered favorable characteristics for probiotic application. Histamine and tyramine, the biogenic amines of primary safety concern, were not detected in any strain, although low levels of tryptamine were observed in *P. pentosaceus* KCA007 and serotonin in *L. brevis* KGK002. Taken together, the complementary phenotypic and genomic safety data support compliance of *P. pentosaceus* TAP041 with established safety criteria for probiotic application.

Whole-genome analysis of *P. pentosaceus* TAP041 provided complementary insight into the functional potential and genomic background of the strain. The genome size (1,785,489 bp), GC content (37.2%), and overall coding density were consistent with previously characterized *P. pentosaceus* strains isolated from fermented food sources (Kompramool et al. 2024), supporting the taxonomic assignment and suggesting a genomically stable chromosomal architecture. Functional annotation revealed a genomic configuration enriched in carbohydrate transport and metabolism (COG category G; *n* = 136) and genetic information processing pathways, reflecting the metabolic adaptability characteristic of lactic acid bacteria. The abundance of carbohydrate-active enzymes, including 37 glycoside hydrolases and 38 glycosyltransferases, suggests a broad capacity to utilize diverse carbohydrate substrates, which may support persistence and metabolic activity in the nutrient-variable environment of the gastrointestinal tract. While these traits are not directly linked to the cellular stress responses examined in the present study, they provide a physiological basis for host colonization and metabolic activity relevant to probiotic function. More specifically, the genome encoded a suite of antioxidant defense components, including NADH peroxidase, thiol peroxiredoxin, glutathione disulfide reductase (glr), and the peroxide-responsive transcriptional regulator PerR, which collectively constitute a coordinated redox defense network characteristic of stress-adapted Gram-positive bacteria (Papadimitriou et al. 2016). The presence of these elements is consistent with the antioxidant activity observed in TAP041 CFS in the present study, and analogous antioxidant systems in probiotic LAB have been associated with modulation of host redox balance in vivo (Wang et al. 2017) although host-level antioxidant effects were not directly assessed here. Glyoxalase-related genes, including lactoylglutathione lyase, hydroxyacylglutathione hydrolase (gloB), and D-lactate dehydratase, were also identified, providing a genomic basis for the MGO-detoxification capacity previously demonstrated for this strain (Lee and Park 2024) and offering mechanistic plausibility for the cellular protection observed under MGO-induced stress conditions (Chakraborty et al. 2015; Frandsen and Narayanasamy 2017). Finally, genes encoding riboflavin biosynthesis enzymes (riboflavin synthase, riboflavin kinase, FMN/FAD metabolism genes, and the riboflavin transporter FmnP) and a comprehensive set of folate pathway genes (including dihydrofolate reductase, dihydrofolate synthase, and the folate transporter FolT) were annotated, indicating predicted biosynthetic potential for vitamins B₂ and B₉. Both riboflavin and folate serve as essential cofactors for neuronal metabolism and have been associated with neurological health (Aragão et al. 2024; Thangaleela et al. 2022), and probiotic-mediated B-vitamin biosynthesis has been reported to influence host micronutrient status in vivo (Perez Visñuk et al. 2022; Rossi et al. 2011). However, actual vitamin production and bioavailability from *P. pentosaceus* TAP041 were not quantified in the present study, and these genomic features are therefore interpreted as indicative of biosynthetic potential at the strain level rather than as evidence of functional vitamin production. Collectively, the genomic features of *P. pentosaceus* TAP041 provide mechanistic plausibility for the observed cellular phenotypes and offer a basis for targeted experimental validation in future studies.

Several limitations of the present study should be acknowledged when interpreting the findings. First, all experiments were conducted under in vitro conditions using SH-SY5Y neuroblastoma cells and primary rat glial cells. Although these models are widely used for mechanistic exploration of neuronal and glial stress responses, they do not recapitulate the complexity of the gut–brain axis, systemic probiotic metabolism, or host–microbiota interactions in vivo. The physiological relevance of the observed effects therefore remains to be established through appropriate animal models of AGE- or LPS-induced neuroinflammation. Second, the functional assays were performed exclusively with CFS rather than live bacterial cells, and the relative contributions of viable bacteria, secreted bioactive metabolites, and any residual medium-derived components to the observed effects could not be clearly distinguished. Third, the mechanistic basis of the observed cellular responses was not directly investigated. Key signaling pathways of potential relevance to the present experimental models, including RAGE-mediated signaling, NF-κB, and MAPK pathways, were not examined, and the observed transcriptional changes in apoptosis-related and neurotrophic markers were not confirmed at the protein-level. Complementary cell death assays, such as Annexin V/PI staining, caspase activity measurements, or LDH release assays, were not performed, and the present findings should therefore be interpreted as transcriptional and phenotypic correlates of cellular protection rather than as direct demonstration of apoptosis inhibition. Fourth, the bioactive composition of TAP041 CFS was only partially characterized. Beyond the antioxidant activity demonstrated by ABTS radical scavenging assays, the specific metabolites responsible for the observed cellular effects—including potential contributions from organic acids, peptides, or extracellular enzymes—were not identified. Direct glyoxalase enzyme activity measurement, B-vitamin quantification, and heat or protease sensitivity testing of the CFS were beyond the scope of the present study and represent essential directions for future characterization. Finally, a formal multi-dose titration was not performed for CFS treatments, and the concentration-dependence of the observed protective effects therefore remains to be systematically evaluated. Collectively, these limitations highlight the preliminary nature of the present findings and underscore the need for mechanistic pathway analyses, protein-level confirmation, comprehensive CFS characterization, and in vivo validation before the translational relevance of *P. pentosaceus* TAP041-derived metabolites in the context of neurodegeneration-related stress can be established.

In summary, the present study demonstrates that cell-free supernatant derived from *P. pentosaceus* TAP041 attenuates glycation- and inflammation-associated stress responses in neuronal and glial cell models in vitro, as evidenced by reduced intracellular ROS accumulation, modulation of apoptosis-related gene expression, suppression of astrocytic inflammatory markers, and partial restoration of neurotrophic marker transcription. These cellular observations are supported at the biochemical level by the antioxidant activity of TAP041 CFS demonstrated in ABTS radical scavenging assays, and at the genomic level by the identification of predicted pathways associated with glyoxalase-mediated detoxification, antioxidant defense, and B-vitamin biosynthesis. Taken together with the strain’s favorable probiotic-related characteristics—including gastrointestinal stress tolerance, intestinal adhesion capacity, and a well-supported safety profile—these findings identify *P. pentosaceus* TAP041 as a candidate strain warranting further investigation in the context of probiotic-based approaches to neurodegeneration-related stress. Nevertheless, the present findings are based exclusively on in vitro models, and mechanistic pathway analyses, protein-level confirmation, and in vivo validation remain essential prerequisites before any translational relevance can be established. Future studies employing animal models of AGE-induced or neuroinflammation-related neurodegeneration, combined with targeted CFS characterization and pathway-specific analyses, will be necessary to determine the physiological significance of the observed effects and to evaluate the potential of *P. pentosaceus* TAP041-derived metabolites as functional ingredients in strategies targeting neurodegeneration-related stress responses.

## Supplementary Information

Below is the link to the electronic supplementary material.

ESM 1 (PDF 300 KB)

## Data Availability

The complete genome sequence of *P. pentosaceus* TAP041 has been deposited in the NCBI database and is accessible under GenBank accession number CP196422, BioProject number PRJNA1295130, and BioSample number SAMN50152112.
