# Supplementary material for: Neuroprotective effects of cell-free supernatant from Pediococcus pentosaceus TAP041 against glycation- and inflammation-associated stress responses
Source: Appl Microbiol Biotechnol. 2026 May 22;110:216. doi: 10.1007/s00253-026-13879-x (PMC13372852; doi:10.1007/s00253-026-13879-x)
Supplement: Supplementary file 1 [file 253_2026_13879_MOESM1_ESM.pdf]

**Applied Microbiology and Biotechnology**

# Neuroprotective effects of *Pediococcus pentosaceus* TAP041 against glycation and inflammation stress

Huijin Jeong, Young Seo Jang, Chaeeun Lee, Hak-Jong Choi, Young-Seo Park*

*Corresponding authors: Young-Seo Park

Department of Food Science and Biotechnology, Gachon University, Seongnam 13120, Republic of Korea

E-mail: ypark@gachon.ac.kr

**Table S1 Primer sequences used for RT-qPCR analysis in glial cells**

| Gene | Direction | Sequence (5′–3′) |
|---|---|---|
| IL-6 | Forward | CACGGCCTTCCCTACTTCAC |
| | Reverse | CTGCAAGTGCATCATCGTTGT |
| TNF-α | Forward | TCGTAGCAAACCACCAAGTG |
| | Reverse | CCTTGAAGAGAACCTGGGAG |
| GAPDH | Forward | GTGTTCCTACCCCCAATGTGT |
| | Reverse | ATTGTCATACCAGGAAATGAGCTT |

**Table S2 PCR amplification conditions in glial cells**

| Step | Temperature (℃) | Time | Cycles |
|---|---|---|---|
| Initial denaturation | 95 | 15 min | 1 |
| Denaturation | 95 | 10 s | 40 |
| Annealing | 58 | 15 s | 40 |
| Extension | 72 | 15 s | 40 |

**Table S3 Primer sequences used for RT-qPCR analysis in SH-SY5Y cells**

| Gene | Direction | Sequence (5′–3′) |
|---|---|---|
| BDNF | Forward | TGACCATCCTTTTCCTTACT |
| | Reverse | GCCACCTTGTCCTCGGAT |
| TH | Forward | GAGGAGAAGGAGGGGAAG |
| | Reverse | ACTCAAACACCTTCACAGCT |
| Bcl-2 | Forward | CGGCTGAAGTCTCCATTAGC |
| | Reverse | CCAGGGAAGTTCTGGTGTGT |
| Bax | Forward | GTGGTTGCCCTCTTCTACTTTGC |
| | Reverse | GAGGACTCCAGCCACAAAGATG |
| GAPDH | Forward | GAGTCAACGGATTTGGTCGT |
| | Reverse | GACAAGCTTCCCGTTCTCAG |

**Table S4 PCR amplification conditions in SH-SY5Y cells**

| Step | Temperature (℃) | Time | Cycles |
|---|---|---|---|
| Initial denaturation | 95 | 15 min | 1 |
| Denaturation | 95 | 10 s | 40 |
| Annealing | 60 | 20 s | 40 |
| Extension | 72 | 30 s | 40 |

**Table S5** E-test for the determination of antibiotic resistance of selected strains

| Antibiotics | EFSA cutoff value (µg/mL) | | Minimum inhibitory concentration (µg/mL) | | |
|---|---|---|---|---|---|
| | *Pediococcus* | *Lactobacillus* obligate heterofermentative | TAP041 | KCA007 | KGK002 |
| Ampicillin | 4 | 2 | 0.25 | 0.19 | 0.75 |
| Vancomycin | n.r. | n.r. | − | − | − |
| Gentamycin | 16 | 16 | 3 | 2 | 0.25 |
| Kanamycin | 64 | 32 | 16 | 6 | 4 |
| Streptomycin | 64 | 64 | 8 | 8 | 4 |
| Erythromycin | 1 | 1 | 0.125 | 0.19 | 0.016 |
| Clindamycin | 1 | 1 | 0.016 | 0.016 | 0.125 |
| Tetracycline | 8 | 8 | 1.5 | 2 | 2 |
| Chloramphenicol | 4 | 4 | 2 | 1.5 | 1 |

n.r., not requires; −, not detected.

**Table S6** Biogenic amine content in the culture supernatant of strains

| Strain | Biogenic amine (ppm) | | | | | | |
|---|---|---|---|---|---|---|---|
| | Agmatine | Histamine | β-Phenylethylamine | Serotonin | Spermidine | Tryptamine | Tyramine |
| TAP041 | − | − | − | − | − | − | − |
| KCA007 | − | − | − | − | − | 9.5 | − |
| KGK002 | − | − | − | 89.8 | − | − | − |

−, not detected.

**Table S7** Enzymatic activity of strains as determined by API ZYM assay

| Enzyme | TAP041 | KCA007 | KGK002 |
|---|---|---|---|
| Alkaline phosphatase | − | − | − |
| Esterase (C4) | − | − | + |
| Esterase lipase (C8) | − | − | − |
| Lipase (C14) | − | − | − |
| Leucine arylamidase | + | + | + |
| Valine arylamidase | + | + | + |
| Cystine arylamidase | − | − | − |
| Trypsin | − | − | − |
| α-Chymotrypsin | − | − | − |
| Acid phosphatase | + | − | + |
| Naphthol-AS-Bl-phosphohydrolase | + | + | + |
| α-Galactosidase | − | − | + |
| β-Galactosidase | + | + | + |
| **β-Glucuronidase** | **−** | **−** | **−** |
| α-Glucosidase | − | − | + |
| β-Glucosidase | + | + | + |
| N-Acetyl-β-glucosaminidase | + | + | − |
| α-Mannosidase | − | − | − |
| α-Fucosidase | − | − | − |

+, indicates positive enzymatic activity; −, indicates no detectable activity.

**Table S8** Functional annotation of genes involved in MGO detoxification and antioxidant pathways in the genome of *P. pentosaceus* TAP041

| Function | Location | Length | Product |
|---|---|---|---|
| Glyoxalase system | 92839..93699 | 861 | 2,5-Didehydrogluconate reductase (2-dehydro-L-gulonate-forming) |
| | 132327..133343 | 1017 | L-glyceraldehyde 3-phosphate reductase |
| | c(421225..421599) | 375 | Lactoylglutathione lyase |
| | c(423318..424169) | 852 | Glycerol 2-dehydrogenase (NADP(+)) |
| | 571804..572481 | 678 | D-lactate dehydratase |
| Glutathione metabolic pathway | 93720..94763 | 1044 | S-(hydroxymethyl)glutathione dehydrogenase |
| | c(174528..175862) | 1335 | Glutathione-disulfide reductase |
| | 260312..261646 | 1335 | Glutathione-disulfide reductase |
| | c(421225..421599) | 375 | Lactoylglutathione lyase |
| | 443980..445053 | 1074 | Glutathione import ATP-binding protein GsiA |
| | 571804..572481 | 678 | D-lactate dehydratase |
| | 766558..766977 | 420 | Hydroxyacylglutathione hydrolase |
| | c(1284762..1285058) | 297 | Hydroxyacylglutathione hydrolase |
| | 1652122..1652595 | 474 | Lactoylglutathione lyase |
| Antioxidant pathway | 26055..26849 | 795 | 3-Oxoacyl-[acyl-carrier-protein] reductase |
| | 46404..47153 | 750 | Dehydrogenase/reductase SDR family protein 7-like |
| | 299116..300456 | 1341 | NADH peroxidase |
| | c(382605..383045) | 441 | Peroxide-responsive repressor PerR |
| | 456075..456629 | 555 | DNA protection during starvation protein |
| | 524431..525453 | 1023 | Glyceraldehyde-3-phosphate dehydrogenase (phosphorylating) |
| | c(580007..580600) | 594 | 8-Oxo-dGTP diphosphatase |
| | 661954..662727 | 774 | Chloride peroxidase |
| | c(1238185..1238676) | 492 | Peroxiredoxin |
| | c(1286497..1287801) | 1305 | Fumarate reductase (quinol) |
| | c(1287798..1288430) | 633 | Fumarate reductase (quinol) |
| | c(1548871..1550241) | 1371 | NADH peroxidase |
| | c(1576355..1577701) | 1347 | NADH peroxidase |

**Table S9** Distribution of genes related to riboflavin, FMN, FAD, and folate metabolism in *Pediococcus pentosaceus* TAP041

| Function | Product | TAP041 |
|---|---|---|
| Riboflavin | Riboflavin kinase | 1 |
| | Riboflavin synthase | 1 |
| FMN | FMN hydrolase | 1 |
| | FMN reductase (NAD(P)H) | 1 |
| | Riboflavin transporter FmnP | 1 |
| FAD | FAD:protein FMN transferase | 1 |
| Folate | 5-Formyltetrahydrofolate cyclo-ligase | 2 |
| | Dihydrofolate reductase | 2 |
| | Dihydrofolate synthase | 1 |
| | Folate transporter FolT | 1 |
| | Formate--tetrahydrofolate ligase | 1 |
| | Methylenetetrahydrofolate dehydrogenase (NADP (+)) | 1 |

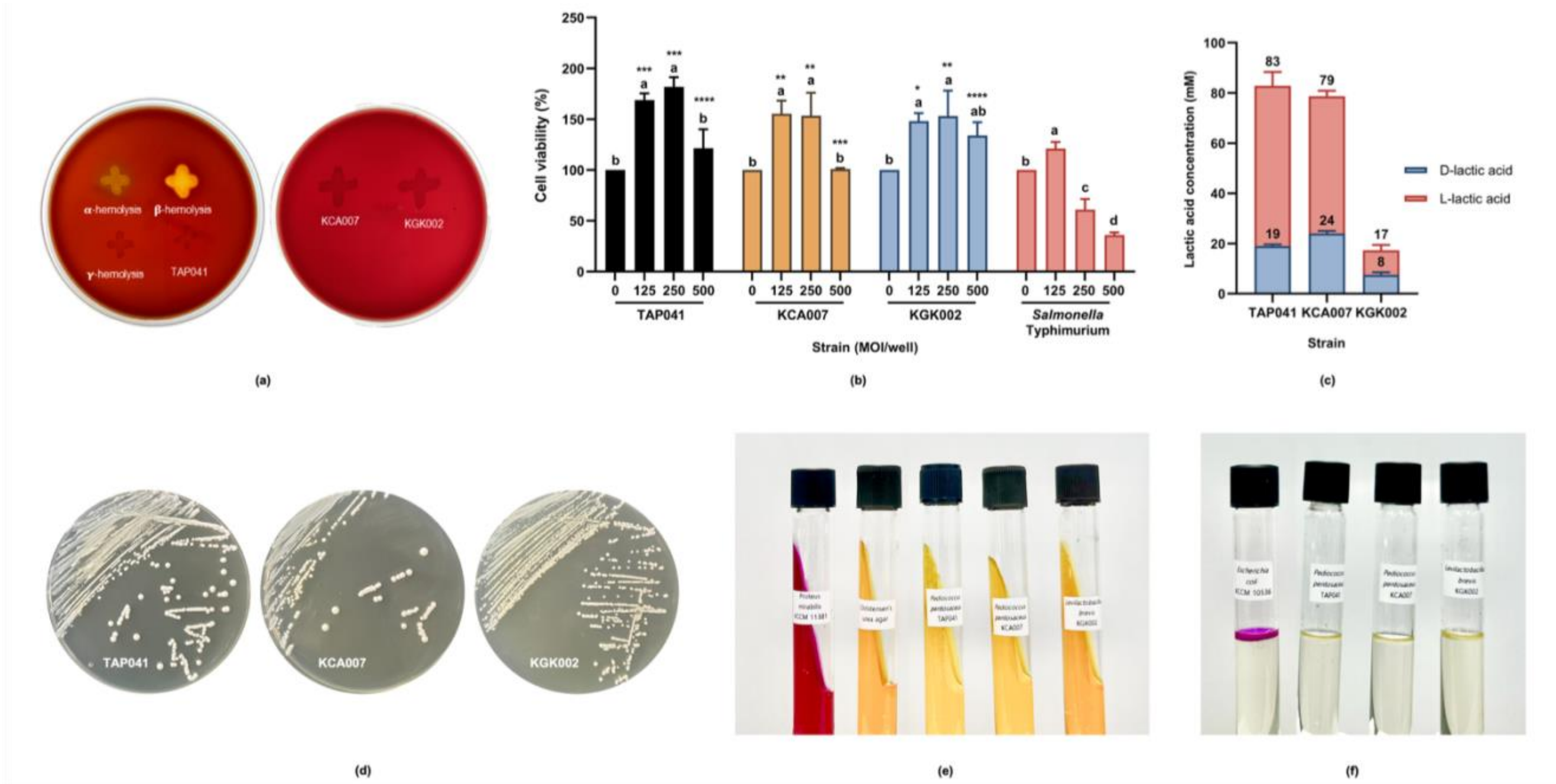


**Fig. S1 Safety assessment of probiotic strains** (a) Hemolytic activity and (b) cytotoxic effects on intestinal Caco-2 cells. Data are presented as mean ± SD (n = 3). Statistical significance was determined using one-way ANOVA followed by Tukey's post hoc test. Different letters indicate statistically significant differences at $p < 0.05$. Significance levels are denoted as follows: $p \leq 0.05$ (*), $p \leq 0.01$ (**), $p \leq 0.001$ (***), and $p \leq 0.0001$ (****), indicating differences between the tested strains and *Salmonella* Typhimurium at the same concentration. (c) D-lactic acid production, (d) bile salt hydrolase (BSH) activity determined by bile salt deconjugation, (e) urease activity, and (f) indole production from tryptophan metabolism were evaluated as additional safety indicators
