# Supplementary material for: Neuroprotective effects of cell-free supernatant from Pediococcus pentosaceus TAP041 against glycation- and inflammation-associated stress responses
Source: Appl Microbiol Biotechnol. 2026 May 22;110(1):216. doi: 10.1007/s00253-026-13879-x (PMC13372852; doi:10.1007/s00253-026-13879-x)
Supplement: Supplementary file 1 — (PDF 300 KB) [file 253_2026_13879_MOESM1_ESM.pdf]

Applied Microbiology and Biotechnology

Neuroprotective effects of *Pediococcus pentosaceus* TAP041 against glycation and inflammation stress

Huijin Jeong, Young Seo Jang, Chaeeun Lee, Hak-Jong Choi, Young-Seo Park*

*Corresponding authors: Young-Seo Park

Department of Food Science and Biotechnology, Gachon University, Seongnam 13120, Republic of Korea

E-mail: ypark@gachon.ac.kr

Table S1 Primer sequences used for RT-qPCR analysis in glial cells

Gene	Direction	Sequence (5′–3′)
IL-6	Forward	CACGGCCTTCCCTACTTCAC
	Reverse	CTGCAAGTGCATCATCGTTGT
TNF-α	Forward	TCGTAGCAAACCACCAAGTG
	Reverse	CCTTGAAGAGAACCTGGGAG
GAPDH	Forward	GTGTTCCTACCCCCAATGTGT
	Reverse	ATTGTCATACCAGGAAATGAGCTT

Table S2 PCR amplification conditions in glial cells

Step	Temperature (℃)	Time	Cycles
Initial denaturation	95	15 min	1
Denaturation	95	10 s	40
Annealing	58	15 s	40
Extension	72	15 s	40

Table S3 Primer sequences used for RT-qPCR analysis in SH-SY5Y cells

Gene	Direction	Sequence (5′–3′)
BDNF	Forward	TGACCATCCTTTTCCTTACT
	Reverse	GCCACCTTGTCCTCGGAT
TH	Forward	GAGGAGAAGGAGGGGAAG
	Reverse	ACTCAAACACCTTCACAGCT
Bcl-2	Forward	CGGCTGAAGTCTCCATTAGC
	Reverse	CCAGGGAAGTTCTGGTGTGT
Bax	Forward	GTGGTTGCCCTCTTCTACTTTGC
	Reverse	GAGGACTCCAGCCACAAAGATG
GAPDH	Forward	GAGTCAACGGATTTGGTCGT
	Reverse	GACAAGCTTCCCGTTCTCAG

Table S4 PCR amplification conditions in SH-SY5Y cells

Step	Temperature (℃)	Time	Cycles
Initial denaturation	95	15 min	1
Denaturation	95	10 s	40
Annealing	60	20 s	40
Extension	72	30 s	40

Table S5 E-test for the determination of antibiotic resistance of selected strains

Antibiotics	EFSA cutoff value (μg/mL)		Minimum inhibitory concentration (μg/mL)		
	Pediococcus	*Lactobacillus* obligate heterofermentative	TAP041	KCA007	KGK002
Ampicillin	4	2	0.25	0.19	0.75
Vancomycin	n.r.	n.r.	−	−	−
Gentamycin	16	16	3	2	0.25
Kanamycin	64	32	16	6	4
Streptomycin	64	64	8	8	4
Erythromycin	1	1	0.125	0.19	0.016
Clindamycin	1	1	0.016	0.016	0.125
Tetracycline	8	8	1.5	2	2
Chloramphenicol	4	4	2	1.5	1

n.r., not requires; −, not detected.

Table S6 Biogenic amine content in the culture supernatant of strains

Strain	Biogenic amine (ppm)						
	Agmatine	Histamine	β-Phenylethylamine	Serotonin	Spermidine	Tryptamine	Tyramine
TAP041	−	−	−	−	−	−	−
KCA007	−	−	−	−	−	9.5	−
KGK002	−	−	−	89.8	−	−	−

−, not detected.

Table S7 Enzymatic activity of strains as determined by API ZYM assay

Enzyme	TAP041	KCA007	KGK002
Alkaline phosphatase	−	−	−
Esterase (C4)	−	−	+
Esterase lipase (C8)	−	−	−
Lipase (C14)	−	−	−
Leucine arylamidase	+	+	+
Valine arylamidase	+	+	+
Cystine arylamidase	−	−	−
Trypsin	−	−	−
α-Chymotrypsin	−	−	−
Acid phosphatase	+	−	+
Naphthol-AS-Bl-phosphohydrolase	+	+	+
α-Galactosidase	−	−	+
β-Galactosidase	+	+	+
**β-Glucuronidase**	**−**	**−**	**−**
α-Glucosidase	−	−	+
β-Glucosidase	+	+	+
N-Acetyl-β-glucosaminidase	+	+	−
α-Mannosidase	−	−	−
α-Fucosidase	−	−	−

+, indicates positive enzymatic activity; −, indicates no detectable activity.

Table S8 Functional annotation of genes involved in MGO detoxification and antioxidant pathways in the genome of *P. pentosaceus* TAP041

Function	Location	Length	Product
Glyoxalase system	92839..93699	861	2,5-Didehydrogluconate reductase (2-dehydro-L-gulonate-forming)
	132327..133343	1017	L-glyceraldehyde 3-phosphate reductase
	c(421225..421599)	375	Lactoylglutathione lyase
	c(423318..424169)	852	Glycerol 2-dehydrogenase (NADP(+))
	571804..572481	678	D-lactate dehydratase
Glutathione metabolic pathway	93720..94763	1044	S-(hydroxymethyl)glutathione dehydrogenase
	c(174528..175862)	1335	Glutathione-disulfide reductase
	260312..261646	1335	Glutathione-disulfide reductase
	c(421225..421599)	375	Lactoylglutathione lyase
	443980..445053	1074	Glutathione import ATP-binding protein GsiA
	571804..572481	678	D-lactate dehydratase
	766558..766977	420	Hydroxyacylglutathione hydrolase
	c(1284762..1285058)	297	Hydroxyacylglutathione hydrolase
	1652122..1652595	474	Lactoylglutathione lyase
Antioxidant pathway	26055..26849	795	3-Oxoacyl-[acyl-carrier-protein] reductase
	46404..47153	750	Dehydrogenase/reductase SDR family protein 7-like
	299116..300456	1341	NADH peroxidase
	c(382605..383045)	441	Peroxide-responsive repressor PerR
	456075..456629	555	DNA protection during starvation protein
	524431..525453	1023	Glyceraldehyde-3-phosphate dehydrogenase (phosphorylating)
	c(580007..580600)	594	8-Oxo-dGTP diphosphatase
	661954..662727	774	Chloride peroxidase
	c(1238185..1238676)	492	Peroxiredoxin
	c(1286497..1287801)	1305	Fumarate reductase (quinol)
	c(1287798..1288430)	633	Fumarate reductase (quinol)
	c(1548871..1550241)	1371	NADH peroxidase
	c(1576355..1577701)	1347	NADH peroxidase

Table S9 Distribution of genes related to riboflavin, FMN, FAD, and folate metabolism in *Pediococcus pentosaceus* TAP041

Function	Product	TAP041
Riboflavin	Riboflavin kinase	1
	Riboflavin synthase	1
FMN	FMN hydrolase	1
	FMN reductase (NAD(P)H)	1
	Riboflavin transporter FmnP	1
FAD	FAD:protein FMN transferase	1
Folate	5-Formyltetrahydrofolate cyclo-ligase	2
	Dihydrofolate reductase	2
	Dihydrofolate synthase	1
	Folate transporter FolT	1
	Formate--tetrahydrofolate ligase	1
	Methylenetetrahydrofolate dehydrogenase (NADP (+))	1

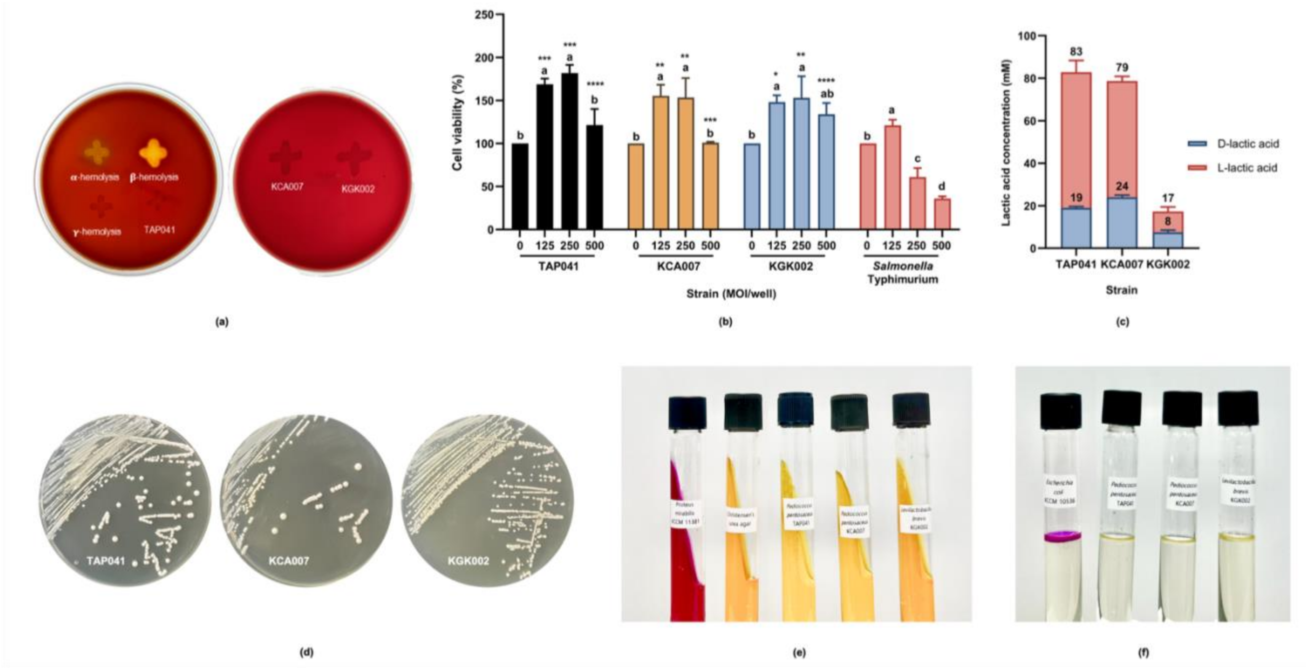

Fig. S1 Safety assessment of probiotic strains (a) Hemolytic activity and (b) cytotoxic effects on intestinal Caco-2 cells. Data are presented as mean ± SD (n = 3). Statistical significance was determined using one-way ANOVA followed by Tukey's post hoc test. Different letters indicate statistically significant differences at $p < 0.05$. Significance levels are denoted as follows: $p \leq 0.05$ (*), $p \leq 0.01$ (**), $p \leq 0.001$ (***), and $p \leq 0.0001$ (****), indicating differences between the tested strains and *Salmonella* Typhimurium at the same concentration. (c) D-lactic acid production, (d) bile salt hydrolase (BSH) activity determined by bile salt deconjugation, (e) urease activity, and (f) indole production from tryptophan metabolism were evaluated as additional safety indicators
